# Phytochemistry, Biological Activities, Molecular Mechanisms, and Toxicity of Saffron (*Crocus sativus* L.): A Comprehensive Overview

**DOI:** 10.3390/antiox14121433

**Published:** 2025-11-28

**Authors:** Anas Ziani, Oussama Bekkouch, Sabir Ouahhoud, Sanae Baddaoui, Soufiane Ben’Mbarek, Ayoub Bekkouch, Amine Khoulati, Bassem Jaouadi, Jinwon Choi, Min Choi, Hyo Jeong Kim, Redouane Benabbes, Abdeslam Asehraou, Moon Nyeo Park, Bonglee Kim, Ennouamane Saalaoui

**Affiliations:** 1 Laboratory of Bioresources, Biotechnology, Ethnopharmacology and Health, Faculty of Sciences, University Mohammed the First, P.O. Box 524, Oujda 60000, Morocco; anas.ziani@ump.ac.ma (A.Z.); oussamabekkouch@hotmail.fr (O.B.); sanae.baddaoui@ump.ac.ma (S.B.); benmbareksoufiane2019@gmail.com (S.B.); khoulati_amine1718@ump.ac.ma (A.K.); r.benabbes@ump.ac.ma (R.B.); a.asehraou@ump.ac.ma (A.A.); e.saalaoui@ump.ac.ma (E.S.); 2 Higher Institute of Nursing Professions and Health Techniques, Oujda 60000, Morocco; 3 Faculty of Medicine and Pharmacy, University Sultan Moulay Slimane, Beni Mellal 23000, Morocco; s.ouahhoud@usms.ma; 4 Biology and Health Laboratory (BHL), Faculty of Ibn Tofail University, Kenitra 14000, Morocco; ayoub.bekkouch@uit.ac.ma; 5 Faculty of Medicine and Pharmacy, University Mohammed the First, P.O. Box 524, Oujda 60000, Morocco; 6 Laboratoire des Biotechnologies Microbiennes et Enzymatiques et de Biomolécules (LBMEB), Centre de Biotechnologie de Sfax (CBS), Université de Sfax, Route Sidi Mansour Km 6, B.P. 1177, Sfax 3018, Tunisia; bassem.jaouadi@cbs.rnrt.tn; 7 Department of Pathology, College of Korean Medicine, Kyung Hee University, Seoul 02447, Republic of Korea; wlsdnjsl6888@naver.com (J.C.); chlals2078@nate.com (M.C.); hyojeong25@khu.ac.kr (H.J.K.); mnpark@khu.ac.kr (M.N.P.); 8 Korean Medicine-Based Drug Repositioning Cancer Research Center, College of Korean Medicine, Kyung Hee University, Seoul 02447, Republic of Korea

**Keywords:** *Crocus sativus*, saffron, crocin, safranal, crocetin, biological effects

## Abstract

Saffron (*Crocus sativus* L.), known as the world’s most valuable spice, has long been appreciated for its culinary, medicinal, and cultural significance. In recent years, increasing scientific attention has been directed toward its unique phytochemical profile and wide-ranging therapeutic potential. This review provides a comprehensive synthesis of current evidence regarding saffron’s chemical composition, molecular mechanisms of action, pharmacological activities, and safety aspects. The stigmas of saffron contain a rich array of bioactive constituents, notably crocin, crocetin, picrocrocin, and safranal, which collectively contribute to its antioxidant, anti-inflammatory, immunomodulatory, cardioprotective, hepatoprotective, neuroprotective, anti-obesity, antidiabetic, and anticancer properties. Evidence from in vitro and in vivo models, as well as clinical studies, suggests that saffron primarily exerts its beneficial effects through the modulation of oxidative stress, apoptosis, autophagy, lipid metabolism, and the regulation of key molecular pathways, including the NF-κB, PI3K/Akt/mTOR, and Nrf2/HO-1 pathways. Furthermore, recent advances in nanotechnology-based formulations have demonstrated improved bioavailability and enhanced therapeutic efficacy, thereby opening up promising avenues for clinical applications. While saffron is generally regarded as safe, challenges remain concerning its high cost, limited availability, and variability in quality due to geographic and environmental factors. Collectively, the accumulated body of evidence highlights saffron as a promising natural agent for the prevention and management of chronic diseases. However, further translational and large-scale clinical investigations are needed to fully establish its therapeutic value and optimize its integration into modern pharmacological and nutraceutical strategies.

## 1. Introduction

The current generation is dealing with the triple combination of sedentary lifestyle, unhealthy eating habits, and stress. These variables are having a devastating effect on human health, causing an increasing number of metabolic diseases and epigenetic alterations that can be fatal [1]. It has been shown that oxidative stress is associated with the development of several metabolic illnesses, chronic ailments, and other malignancies [2]. Reactive oxygen species (ROS) generated by the body in limited levels control cell homeostasis, signal transmission, gene expression, and receptor activation [3,4]. An imbalance between the production of reactive oxygen species (ROS) and their elimination leads to oxidative stress, which can be detrimental to all essential cellular structures. To neutralize ROS, the body protect itself with enzymatic antioxidants such as superoxide dismutase (SOD), catalase (CAT), glutathione peroxidase (GPx) and their cofactors (thioredoxin, glutathione) [5]. In addition of many foods include well-known antioxidants such polyphenols, flavonoids, and carotenoids, which have been the subject of much scientific investigation about their potential health benefits [6], including pant-based phenolics known for their antioxidant power, as demonstrated in Moroccan aromatic plants [7].

Vegetables and medicinal aromatic plants (MAPs) are essential sources of bioactive compounds. Due to their powerful antioxidant properties, these compounds may help prevent several chronic illnesses when consumed regularly. They have been linked to a lower risk of diseases connected to oxidative stress, as well as several cardiovascular problems like high cholesterol and hypertension. They also might guard against type II diabetes, particular malignancies, urinary tract infections, and neurological diseases like Alzheimer’s and Parkinson’s. However, their health benefits depend on factors such as the specific type of phenolic compound, the amount consumed, and their bioavailability in the body [8,9].

One spicy, medicinal plant is *Crocus sativus* L. (Iridaceae), also known as saffron, which is renowned for its culinary uses, coloring properties, and historical significance in traditional medicine [10]. Traditional saffron spice is made from the dried stigmas of the *Crocus sativus* L. flower [11]. It is the most expensive spice in the world, hence its nickname, “Red Gold.” The flowers are usually picked by hand in the early morning around mid-October. The stigmas are picked from flowers, dried, and then ground into powder. Approximately 70,000–200,000 flowers are required to produce 1 kg of saffron; each flower weighs between 0.3 and 1.0 g. This helps to explain why saffron stays among the most costly medicinal plants available worldwide [12].

More than 90% of saffron made in the world comes from Iran, while the Herat region of Afghanistan produces about 5–6 tons of saffron each year [12,13]. Other producers include India, Greece, and Morocco, although on a smaller scale. Beyond its culinary prestige, saffron’s high market value and rich phytochemical profile have made it a focus of pharmacological and nutraceutical research [14].

## 2. Methodology

In the present review, a literature-based search covering studies released during the past ten years, was performed to retrieve information on the chemistry, health effects, molecular pharmacology, herb–drug interaction, nanotechnology-based drug delivery, and safety of saffron from accessible online databases, such as Google Scholar, Science direct, Web of Science, Scopus, and PubMed, using the key search terms of ‘*Crocus sativus*’ and chemical constituents, antioxidant, anti-inflammatory, hepatoprotective, cardioprotective, nephron-protective, anti-obesity, neuroprotective, immunomodulatory, anticancer, nanotechnology or toxicity, etc. This review covers those articles that demonstrate the health benefits of saffron and its bioactive compounds. The authors carefully chose and reviewed all the sources included.

## 3. Evolution of Trends in Research with *Crocus sativus*

**Figure 1:**
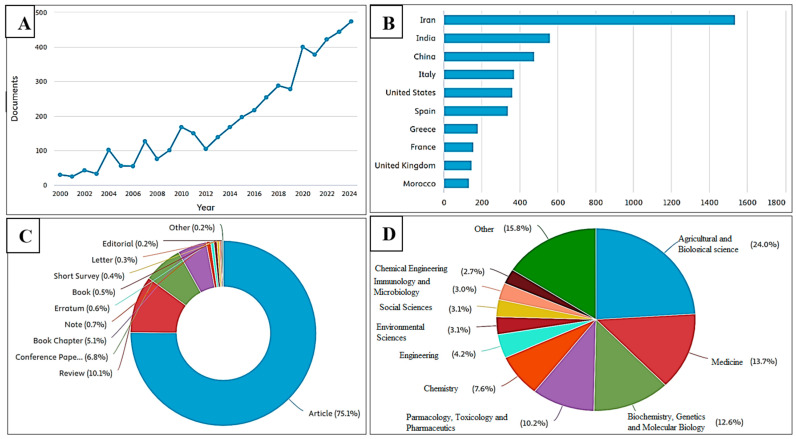
Scientific progress and perspectives on saffron. (**A**) Annual distribution of publications. (**B**) Top 10 research-producing countries. (**C**) Publications shared by document category. (**D**) Percentage breakdown of publications by research domain. The data were collected from the Scopus database in March 2025.

Evidence for the use and cultivation of saffron dates back to ancient times. The earliest indication of Crocus usage by humans was identified in a 50,000-year-old cave painting in present-day Iraq, where pigments derived from saffron were applied. However, *Crocus sativus* L. first appeared in literature much later, around 350–300 BCE, in Historia Plantarum by Theophrastus [15]. The earliest compound to be isolated from *Crocus sativus* L. was safranal, reported in 1922. It was obtained through both acid- and alkali-catalyzed hydrolysis, marking the first documented identification of a saffron constituent [16]. Significant growth is observed after 2016, with a sharp rise from 2019 to 2021, followed by a continued increase, reaching nearly 500 documents by 2024. This pattern indicates a growing and sustained research interest in the scientific community regarding the pharmacological potential of saffron over the past two decades (Figure 1A). The majority of research papers have been produced in Iran, with more than 1500 documents, far surpassing those of any other country. India ranks second, followed by China, which contributes significantly but at less than half the output of Iran. Italy and the United States generate a comparable number of studies, placing them in the mid-range alongside Spain.

In contrast, Greece, France, the United Kingdom, and Morocco account for a smaller share of the total research activity (Figure 1B). The publication landscape is primarily composed of full articles (75.1%), with reviews (10.1%), conference papers (6.8%), and book chapters (5.1%) making up the remainder (Figure 1C). The research covers a wide range of fields, including agricultural and biological sciences, medicine, biochemistry, genetics, molecular biology, pharmacology, toxicology, pharmaceutics, chemistry, engineering, immunology, and several other disciplines (Figure 1D).

## 4. *Crocus sativus* Taxonomy

The family Iridaceae is represented by approximately 70 genera and more than 2030 species [16]; Crocus is a genus within the Iridaceae family. *Crocus sativus* is a medicinal and aromatic plant, and its taxonomic position is as follows (Table 1).

Saffron is a geophyte plant that blooms in autumn and replicates exclusively through underground corms. With a basic chromosome number of x = 8, it is a triploid and sterile plant, relying entirely on vegetative propagation. Under normal conditions, each mother corm typically produces only four to five daughter corms per growing season [17,18].

**Figure 2:**
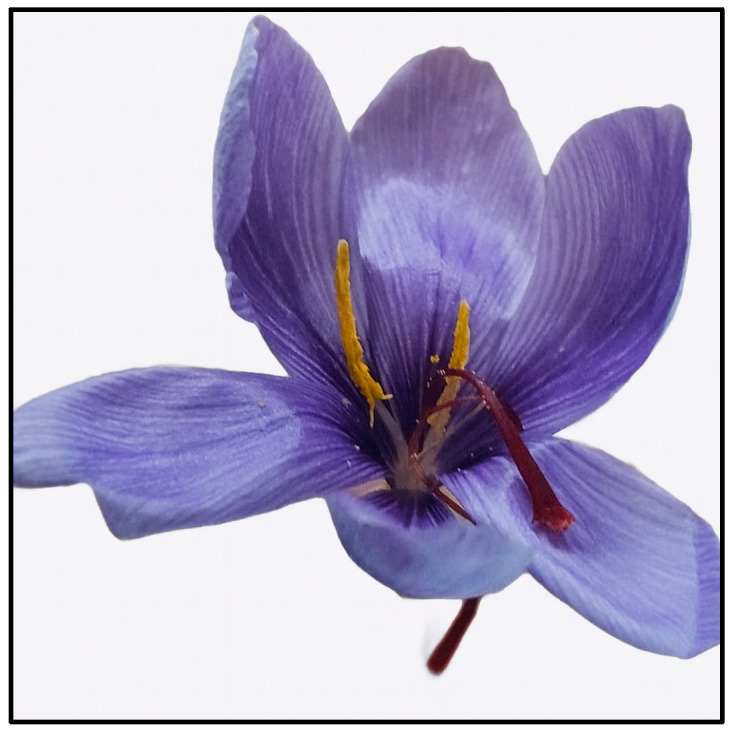
*Crocus sativus* flower. Photograph taken by the authors in the laboratory of Bioresources, Biotechnology, Ethnopharmacology and Health, Faculty of Sciences, University Mohammed the First.

The cultivation of *Crocus sativus* is adapted to arid and semi-arid areas; this plant grows well in areas with rainy spring and autumn, and dry summer. It can also tolerate temperate and subtropical climates with sandy or clay soils [19,20]. Each flower is composed of six tepals, three stamens, and a filiform white style that terminates in a stigma split into three threads (Figure 2). While growing, stigmas change colors from white to intense red. Saffron spice is obtained after drying the stigmas [21,22]. The production of secondary metabolites depends on the geographical origin and environmental conditions such as altitude, temperature, rainfall, irrigation cycles, harvest season, humidity, and soil properties [23,24].

In what is now Iraq, saffron-based pigments have been discovered in 50,000-year-old depictions of prehistoric animals [15,25]. The Sumerians, one of the earliest civilizations (circa 4100–1750 BCE), are believed to have used saffron for its medicinal and therapeutic properties [25]. Its value was not only in its treatment but also in its perceived magical and protective qualities [26]. This early use of saffron highlights its longstanding significance in human history, predating many documented medicinal traditions [27]. Meanwhile, the Phoenicians utilized their expertise in textile production and commerce, enabling them to play a pivotal role in trade, and provided saffron-dyed fabrics to the kings of Assyria [28]. During the 10th century BCE, in ancient Persia, saffron was cultivated in regions such as Darband, Isfahan, and Khorasan [29]. It was destined to be used in textiles, which were then offered as sacred gifts to goddesses during religious rituals. Beyond its spiritual significance, saffron was also prized for its versatility; it was used to dye fabrics, create perfumes, produce medicines, and even enhance bathing rituals.

Ancient Iranians also believed in its therapeutic properties, using it to treat melancholia by scattering it around their beds or mixing it into hot tea, and it was used for its aphrodisiac qualities [29].

Saffron was introduced to Egypt from Crete during the ancient Egyptian period (3100 BC–476 AD). References for its Medicinal uses can be found in the Ebers Papyrus, where it was used to treat eye disorders and regulate menstruation and urinary disorders. Additionally, saffron was utilized to support and ease the process of childbirth [26].

**Figure 3:**
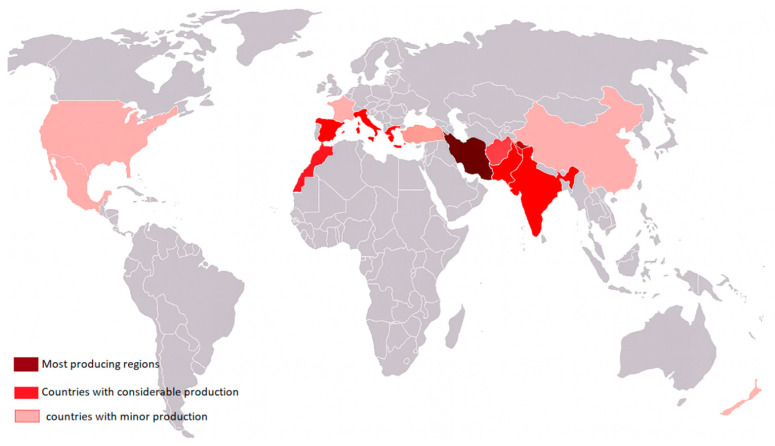
Principal regions of Saffron production worldwide. (original map; [32]).

The introduction of saffron to India is often attributed to ancient Persians, though the precise timeline remains uncertain. Historical accounts suggest that by the 3rd century AD. It was a luxury product reserved for wealthy people; they often used it as a perfume or as an air freshener, and they dyed their robes and even used it just as décor [30]. Its presence in North Africa began around the 9th century, following the Arab–Muslim conquests. Later, during the Moorish occupation, saffron cultivation expanded into Europe, especially in Spain. In France, its introduction is linked to the Crusades between the 11th and 13th centuries, a period during which it was also cultivated in regions such as Germany, Switzerland, and Italy [31]. Currently, the major saffron-producing countries include Iran, Greece, India, Morocco, Spain, and Italy (Figure 3).

## 5. Saffron Natural Bioactive Compounds

The phytochemical composition of saffron is highly rich and diverse, containing more than 150 compounds (volatile and non-volatile) [24], including colored carotenoids such as crocetin and its glycosidic derivatives (crocins). In addition, saffron contains colorless monoterpene aldehydes, volatile compounds like safranal, which is responsible for its distinct aroma, and bitter components like picrocrocin, responsible for its characteristic taste [19,24,33]. The quality and the commercial values of saffron spices depend on the presence of three main bioactives, apocarotenoids (crocin, picro-crocin, safranal) [20]. There are also low levels of lipophilic carotenoids, including lycopene, zeaxanthin, and β-carotene [34]. Flavonoids were also detected in saffron stigmas. A study using HPLC-DAD-ESI-MS/MS, identified tetra-, tri-, and di-hexosylated forms of kaempferol [35].

Furthermore, saffron contains lipophilic and hydrophilic carbohydrates, proteins, amino acids, minerals, mucilage, starch, gum, vitamins, alkaloids, and saponins [36].

### 5.1. Crocin

**Figure 4:**
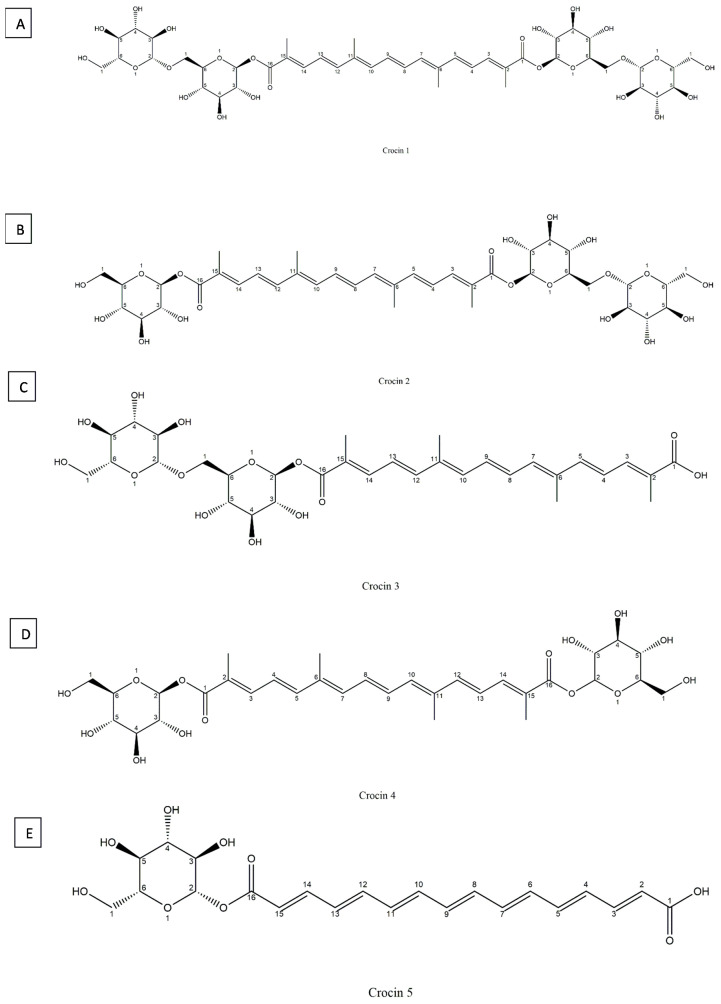
Chemical structures of crocin 1–5, shown as (**A**) crocin 1; (**B**) crocin 2; (**C**) crocin 3; (**D**) crocin 4; and (**E**) crocin 5.

Crocin or 8′-diapocarotene-8,8′-dioic acid, is a rare carotenoid in nature, with a chemical formula: C_44_H_70_O_28_ and molecular weight of 976.96 g/mol, constitutes approximately 6 to16% of saffron’s total dry matter, the percentage varies depending on (variety, growing conditions, and processing methods) [37]. It is a diester of crocetin with gentobiose and is easily soluble in water due to its high glycosyl content. Unlike most carotenoids, which are water-insoluble, crocin is widely used in food and medicine [36,38]. Crocin-1 (α-crocin or digentiobioside crocetin) (Figure 4A), crocin-2 (tricrocin or gentioglucoside crocetin) (Figure 4B), crocin-3 (gentiobioside crocetin) (Figure 4C), crocin-4 (diglucoside crocetin) (Figure 4D), and crocin-5 (glucoside crocetin) (Figure 4E) exhibit remarkable stability under ambient conditions [36].

### 5.2. Crocetin

**Figure 5:**
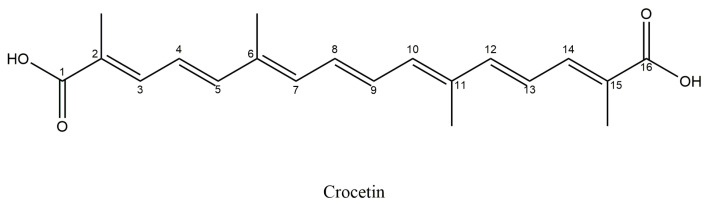
Structural formula of Crocetin.

Crocetin or 8,8′-diapo-8,8′-carotenoic acid (Figure 5) is an amphiphilic carotenoid, with a chemical formula: C_20_H_24_O_4_ and a molecular weight of 328.4 g/mol, that constitutes approximately 14% of saffron’s total dry matter. Crocetins (α-crocetin or crocetin I, crocetin II, β-crocetin, γ-crocetin) are products of the oxidative cleavage of zeaxanthin precursor by specific carotenoid cleavage oxygenases (CCDs) [36,39].

### 5.3. Picrocrocin

**Figure 6:**
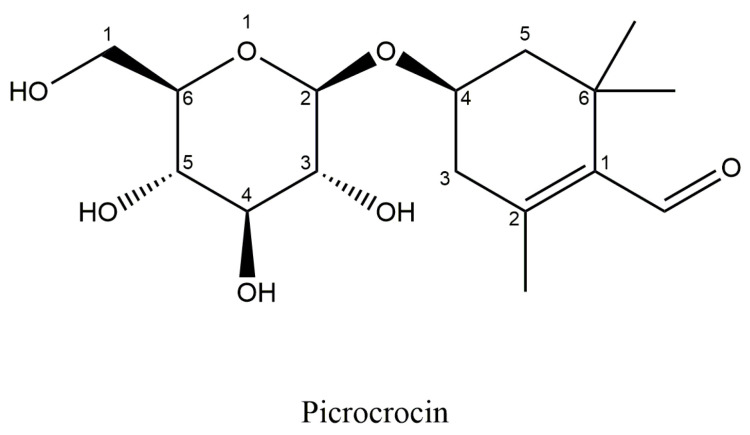
Structural formula of Picrocrocin.

Picrocrocin is a glycoside of safranal (Figure 6), with a chemical formula: C_16_H_26_O_7_ and molecular weight of 330.37 g/mol, is the main crystalline terpene-glucoside of saffron responsible for the bitter flavour of saffron [22,36]. It constitutes approximately 3.7% of the weight of the stigma [36]; in acidic and alkaline conditions, this molecule undergoes hydrolysis, yielding a glucose molecule and an aglycone called 4-hydroxy-β-cyclocitral. The loss of water spontaneously converts 4-hydroxy-β-cyclocitral into dehydro-β-cyclocitral or safranal [36,38,40].

### 5.4. Safranal

**Figure 7:**
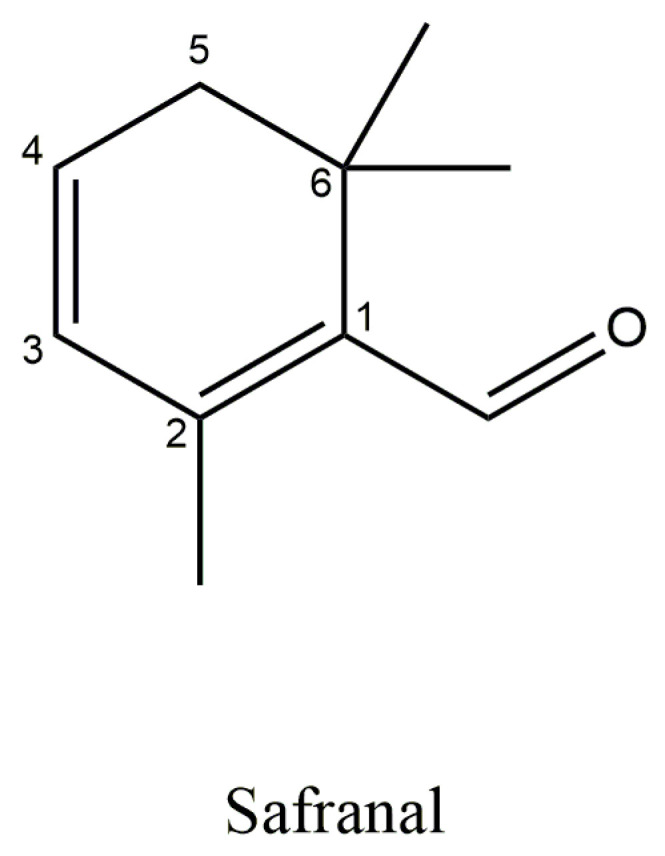
Structural formula of Safranal.

Safranal is the aglycone of picrocrocin (Figure 7), responsible for the aroma and distinctive scent of saffron. This volatile compound accounts for approximately 60% of the total volatile content in stigmas [38,40]. Safranal or 2,6,6-trimethyl-1,3-cyclohexadiene-1-carboxaldehyde is a monoterpene aldehyde with a chemical formula: C_10_H_14_O and molecular weight of 150.21 g/mol [40]. Since safranal is not initially present in fresh *Crocus sativus* stigmas, its concentration in the final product is highly dependent on drying and storage conditions [41].

## 6. Benefits of Saffron Stigmas on Human Health and Disease Conditions

### 6.1. Antioxidant Activity

Antioxidants are essential for preserving cellular homeostasis by neutralizing reactive oxygen species (ROS) and preventing oxidative damage to lipids, proteins, and nucleic acids [42,43]. Oxidative stress, resulting from an imbalance between ROS production and antioxidant defense systems, is usually involved in the pathogenesis of numerous chronic diseases, including cardiovascular disorders, neurodegenerative diseases, diabetes, and cancer. Saffron and its bioactive compounds, antioxidants, can act through various mechanisms, including free radical scavenging, inhibition of lipid peroxidation, metal ion chelation, and modulation of antioxidant enzyme activity [42]. Numerous scientific reports have evaluated saffron and its bioactive molecules as potent antioxidants in both in vitro and in vivo studies.

(a) In vitro activity

A study conducted by Ouahhoud et al. revealed that saffron stigmas exhibited vigorous antiradical activity, with an IC_50_ value of 1554.37 µg/mL. They also showed high reducing power and moderate metal-chelating activity for both copper and iron, with no genotoxicity confirmed by the comet assay. Furthermore, pretreatment with the extracts reduced DNA damage induced by streptozotocin and alloxan in leukocytes, likely through the scavenging of reactive oxygen species (ROS) and metal chelation [44]. A study on Lebanese saffron from different regions confirms the role of phenolics, alongside crocin and safranal, in driving saffron’s antioxidant potential [45]. A different experiment on fortifying yoghurt with saffron (0.125 g/kg) found that it significantly increased the total polyphenol content (TPC), resulting in a marked improvement in antioxidant activity compared to the control [46]. In a separate investigation, the aqueous extract of saffron exhibited high radical scavenging activity, while reducing power assays (FRAP, CUPRAC, and phosphomolybdenum) confirmed its high electron-donating capacity. The same extract also displayed the highest metal-chelating ability.

In an ex vivo colon model, treatment with saffron water extract markedly reduced LPS-induced malondialdehyde (MDA) levels, indicating effective inhibition of lipid peroxidation [47].

(b) In vivo activity

Beyond these in vitro studies, in vivo investigations have provided strong support for saffron’s role in mitigating oxidative damage and preserving tissue function under pathological conditions. Hariri et al. reported that saffron administration against the subacute effects of diazinon reduced liver (AST, ALT, ALP, LDH) and heart injury (CK-MB) markers, thereby restoring enzyme activities to normal levels. Additionally, saffron mitigated lipid peroxidation and enhanced endogenous antioxidant defenses. At a dose of 200 mg/kg, saffron improved hematological parameters (RBC, hemoglobin, hematocrit) altered by DZN, suggesting protection of red blood cells from oxidative damage [48]. In C57BL/6J mice treated orally with crocin-1 (20 and 40 mg/kg for 2 weeks), a significant reduction in ROS levels, inhibition of lipid peroxidation, restoration of antioxidant enzyme activities, and mitochondrial protection via SIRT3-mediated pathways were observed [49].

(c) clinical trials

In a randomized clinical trial on patients with ischemic stroke, Patients received a capsule of saffron (200 mg twice daily) for four days. Saffron was found to restore the activities of key antioxidant enzymes, SOD and CAT, increase GSH levels, enhance GPx and GST activities, and significantly reduce malondialdehyde (MDA) levels [50].in another clinical study, 8 weeks of saffron supplementation in ulcerative colitis (UC) patients increased Total antioxidant capacity, SOD and GPx, while MDA decreased [51].

### 6.2. Anti-Inflammatory Activity

Inflammation is a natural defense mechanism against injury or infection; however, persistent or dysregulated inflammation is associated with various pathophysiological conditions, including cancer, arthritis, cardiovascular diseases, and neurodegenerative disorders [52]. Saffron is well recognized for its potent anti-inflammatory properties, which are attributed to its high content of bioactive compounds. In recent years, numerous experimental models and clinical studies have confirmed this powerful effect. Jeddi et al. investigated the anti-inflammatory effect of crocin in OVA-sensitized mice and found a reduction in IgE levels, indicating suppression of allergic inflammatory responses. It also downregulated NF-κB mRNA and protein expression, thereby blocking a key pro-inflammatory signaling pathway [53]. Samarghandian et al. reported that saffron extract inhibited inflammatory mediators (TNF-α, IL-6) in the aorta, suggesting vascular protection via anti-inflammatory pathways [54]. In response to oral administration of crocin (25 mg/kg) to albino mice, pulmonary inflammation introduced by the ovalbumin was attenuated, with reductions in TNF-α, IL-4, and IL-13, thereby modulating Th2-related immune responses [55]. Another study reported that high-dose crocin administration in mice suppressed atherosclerosis-induced inflammation, primarily by activating the Nrf2 pathway and inhibiting the TLR4/NLRP3 inflammasome signaling cascade [56]. Similar results were reported by Li et al., who found that crocin reduced pro-inflammatory markers (IL-6, iNOS, TNF-α) and increased anti-inflammatory cytokines (IL-10, IL-4, TGF-β). It also inhibited NF-κB p65 expression and nuclear translocation, thereby reducing the activation of downstream inflammatory genes [57]. Additionally, a study reported that crocin suppresses inflammatory signaling by reducing LPS-induced production of the pro-inflammatory cytokines IL-1β, IL-6, and TNF-α in NP cells. It also decreased iNOS expression, thereby limiting nitric oxide–mediated oxidative stress. Furthermore, crocin downregulated catabolic enzymes (MMP-1, MMP-3, MMP-13, ADAMTS-4, and ADAMTS-5), which are typically upregulated during inflammation [58]. In line with these findings, saffron extract (25 and 50 mg/kg) was administered via gastric gavage in rats with adjuvant arthritis. It was found that saffron, particularly at the higher dose, decreased plasma IL-17A and IL-1 levels and downregulated hepatic IL-1 mRNA expression, likely through inhibition of the NF-κB and JNK pathways, and possibly via STAT3 suppression [59]. Clinical investigations in COPD patients have confirmed improvements in pulmonary function tests (PFTs) and 6-min walking distance (6MWD), along with significant reductions in serum levels of IL-6 and TNF-α [60]. Both preclinical and clinical evidence suggest that saffron and its bioactive constituents, particularly crocin and safranal, exhibit significant anti-inflammatory effects.

### 6.3. Immunomodulatory Effects

The immune system, a complex network of cells, tissues, and molecules, defends the body against pathogens such as bacteria and viruses, and maintains homeostasis [57]. Among various natural substances that possess immunomodulatory properties, saffron (*Crocus sativus* L.) has gained attention for its potential benefits. A study using Balb/c mice to investigate the immunotoxic effects of safranal found that, at the tested doses, safranal did not alter immune cell counts, cytokine production, or the histopathology of the spleen and bone marrow, and was considered safe for the mice’s immune system [61]. In another experimental model, crocin (10 mg/kg for 4 weeks) was administered to domestic short-haired cats, which enhanced humoral immunity, as evidenced by a marked increase in IgA and IgG levels, particularly after day 20. IgG levels, once peaked, remained elevated, indicating sustained immune stimulation [62]. Yousefi and colleagues reported that, in vitro, crocin and crocetin at low concentrations enhance the frequency of regulatory T cells, promote the production of anti-inflammatory cytokines (e.g., IL-10, TGF-β), and reduce pro-inflammatory mediators such as TNF-α, IL-6, IL-1β, and IL-17. Crocin showed slightly greater efficacy than crocetin in lowering IFN-γ and IL-4 levels, whereas crocetin more effectively increased IL-10 and TGF-β.

In contrast, high concentrations appeared to be cytotoxic, reducing the viability of MSCs [63]. Tolba and colleagues reported that saffron supplementation in diets (0.5 and 1.5 g/kg) for 12 weeks to Oreochromis niloticus, particularly at 0.5 g/kg, enhanced several immune-related parameters, including higher serum total protein, globulin, lysozyme activity, and IgM levels compared to controls, indicating improved humoral and innate immune responses. Additionally, immunohistochemical analysis revealed decreased expression of the pro-inflammatory cytokine TNF-α and the macrophage marker CD68, supporting saffron’s anti-inflammatory and immunomodulatory roles. At the same time, the higher dose caused mild histological alterations in the gills, liver, and spleen [64]. A clinical trial involving patients with sepsis was conducted, following saffron supplementation (100 mg/day for 7 days), which resulted in a significant improvement in clinical, inflammatory, and hematological parameters [65].

### 6.4. Anti-Cancer Activity

Owing to their ability to modulate various key parameters involved in cancer treatment, saffron and its bioactive molecules could serve as substitutes for certain drugs and as adjuvants to chemotherapy, thereby enhancing therapeutic outcomes. Across diverse cell models lines including A172 glioblastoma, rhabdomyosarcoma TE671 [66], HeLa cells [67], colorectal cancer colo-205 [68], oral squamous carcinoma SCC-25 [69], triple-negative breast cancer (HCC70, HCC1806 and CCD1059) [70], epidermoid carcinoma HEp-2 [71], malignant TC-1 and non-malignant COS-7 [72], melanoma B16F10, MCF-7, lung adenocarcinoma A549 and ovarian carcinoma SKOV3 [73], renal carcinoma Kidney Caki-1 and bladder cancer RT4 and RT112 cell lines [74], and Human Prostate Cancer Cells line MDA-PCa-2b [75]. In these models, saffron and its derivate compounds particularly crocin, crocetin, and safranal have consistently been shown to inhibit cancer cell proliferation, induce apoptosis, interfere with cell cycle progression, and modulate oxidative stress and inflammatory pathways. For example, Jiang et al. reported that crocin exhibits selective cytotoxicity, inhibiting cancer cell proliferation while being non-toxic or even cytoprotective to diverse healthy cells. Mechanically, its effects involve the activation of the mitochondrial apoptotic pathway (BAX/BID↑, BCL-2↓), caspase engagement, downregulation of c-Myc/MYCN, suppression of the PI3K/AKT, MAPK, and STAT3/JAK signaling pathways, and the frequent induction of G0/G1 cell cycle arrest [67]. Using the comet assay and annexin V/PI staining, Zhang et al. documented that safranal exhibits potent anti-colon cancer activity in COLO-205 cells. Apoptosis proceeds via the mitochondrial pathway, as evidenced by increased Bax expression, decreased Bcl-2 levels, ROS accumulation, and loss of mitochondrial membrane potential. Safranal also induces G2/M cell cycle arrest and suppresses the PI3K/AKT/mTOR signaling axis [68]. The digentiobiosyl ester of crocetin, or α-crocin, was found to disrupt mitosis by preventing proper microtubule assembly, causing multipolar spindles, chromosome distortion, and centrosome fragmentation, similar to vinblastine or paclitaxel. It inhibits tubulin polymerization at low concentrations and induces tubulin aggregation at higher doses [70]. Building on these varied in vitro findings, researchers have worked to uncover how saffron compounds produce their anticancer effects and to confirm their potential through studies in animals and clinical trials. A recent study evaluated the in vitro cytotoxic potential of saffron alongside its in vivo pre-clinical toxicity profile in rats, providing insights into both its anticancer efficacy and safety. Histological analysis of liver, kidney, spleen, lung, and heart, shows that saffron supplementation (300 mg/kg for 60 days) revealed no pathological abnormalities, indicating its safety for normal tissues.

Additionally, it exhibits selective cytotoxicity against HEp-2 cancer cells while sparing normal Vero cells [71]. Another in vivo study led by Khavari et al. has shown that crocin has a potent anti-tumor effect, with 100% of treated mice remaining tumor-free [72]. A recent study examined the combination of saffron and immunotherapy drugs. It was shown that the body mass of mice increased after the initial loss caused by tumor modeling. In addition, tumor size and weight decreased, and serum IL-17 levels were reduced [1]. RT-PCR and immunohistochemistry further confirmed decreased expression of tumor-associated mRNA and PD-L1 in tumor tissues. Moreover, Flow cytometry showed an increased proportion of CD8^+^ T cells [2], indicating more vigorous cytotoxic immune activity [3,76]. Finally, a study using saffron nanoliposome significantly suppressed tumor growth from days 20 to 30, with the 300 mg/kg dose showing the most significant effect [77]. In summary, the available reports demonstrate that saffron and its major constituents, including crocin, crocetin, and safranal, exert potent anticancer effects through multiple molecular mechanisms as illustrated in Table 2. These findings suggest that saffron-derived compounds hold considerable promise as adjuvant or alternative agents in the prevention and management of various cancers.

### 6.5. Protection Against Neurological Disorders

Saffron and its primary bioactive compounds have been linked to improvements in various neurological disorders. The main objective of this section is to critically review and update the current evidence on the neuroprotective effects of saffron, based on a selection of in vitro, in vivo, and human clinical studies that have evaluated the efficacy of saffron or its key constituents. Preclinical studies have demonstrated their effectiveness in diverse models of neurodegenerative and neuropsychiatric disorders. At the same time, clinical trials have provided promising evidence in conditions such as Alzheimer’s disease, Parkinson’s disease, schizophrenia, and multiple sclerosis (Table 3).

Crocin, the most abundant bioactive compound in saffron, has been shown to exert neuroprotective effects and improve outcomes in Post-Traumatic Stress Disorder (PTSD) models. When combined with extinction learning, crocin increased pain threshold, reduced PTSD-like behaviors, and elevated brain-derived neurotrophic factor (BDNF) levels [88]. A study reported that saffron administration to rats subjected to maternal social isolation from PND reversed memory impairments, reduced locomotor activity, pain sensitivity, and GSK-3β overexpression in adolescents, and partially improved anxiety and depression in adults. Moreover, crocin reduced hippocampal GSK-3β expression, which appears to contribute to the cognitive and mood impairments induced by maternal stress [89]. Salem and colleagues reported that intraperitoneal administration of crocin to rmTBI mice reduced brain inflammatory cytokines, lipid peroxidation, and motor deficits without histological damage. It also decreased mRNA levels of pro-apoptotic genes (p53, Bax, caspase-3), increased anti-apoptotic Bcl-2, and lowered the Bax/Bcl-2 ratio [90]. It has been reported that crocin supplementation alleviates anxiety and depressive like behaviors induced by unpredictable chronic mild stress (UCMS) in rats. Crocin improved behavioral outcomes in the Elevated Plus Maze (EPM), Forced Swim Test (FST), and Open Field test (OF) tests, decreased corticosterone, restored antioxidant defenses, and reduced oxidative damage. It also downregulated IL-6 and TNF-α while increasing IL-10, indicating an anti-inflammatory effect [91]. Also, BDNF levels were elevated in crocin-treated rats, supporting its neuroprotective role [91].

In Pb-induced neurotoxicity in Meriones strains, treatment with saffron (50 mg/kg orally) prevented Pb-induced locomotor deficits and mitigated dopaminergic disruptions, likely due to its high antioxidants and neuroprotective mechanism [92]. Furthermore, in a rotenone-induced Drosophila Parkinson’s disease model, saffron methanolic extract and crocin significantly reduced oxidative stress markers (ROS, hydroperoxides, NO) and enhanced antioxidant defenses (GSH, TR, SOD, CAT, GST). These interventions improved locomotor performance, increased survival rates, and mitigated mitochondrial dysfunction by restoring MTT levels and dopaminergic function. They also normalized acetylcholinesterase activity, indicating protection of cholinergic function [93]. In addition, continuous treatment with crocin (30 mg/kg, intraperitoneally, for 30 days) ameliorated rotenone-induced Parkinson’s disease in rats by activating the PI3K/Akt/mTOR signaling cascade, increasing phosphorylation of PRAS40 and p70S6K, and inhibiting pro-apoptotic proteins (GSK-3β, FoxO3, caspase-9) while enhancing the anti-apoptotic protein Bcl-2. These molecular effects mitigated mitochondrial dysfunction and oxidative stress, thereby limiting the loss of dopaminergic neurons. Histological and biochemical analyses further confirmed reduced neurodegeneration and apoptosis [94].

An ischemic stroke happens when blood flow to a specific region of the brain is suddenly interrupted, leading to impaired cellular function and resulting in either reversible or permanent neurological deficits. Abdel-Rahman and colleagues reported that saffron extract (100 or 200 mg/kg, intraperitoneally, for 3 weeks) conferred protection against ischemic stroke in rats. The treatment reduced infarct damage and attenuated apoptotic markers (BAX, caspase-3). It also significantly increased VEGF expression in ischemic brain tissue, potentially promoting angiogenesis and neuronal survival through the PI3K/Akt pathway [95]. Another study evaluated the effects of safranal on transient focal cerebral ischemia/reperfusion (I/R) injury in rats. Safranal was shown to reduce neurological deficits, neuronal death, and oxidative damage, with the lower dose providing more consistent protection. Its antioxidant action was supported by decreased malondialdehyde (MDA) levels and restored glutathione (GSH) content [96]. Zhang et al. reported the neuroprotective effects of safranal against spinal cord injury in rats. It has been found that the treatment improved locomotor function in a dose-dependent manner, with 100 mg/kg showing optimal efficacy. Safranal reduced aquaporin-4 (AQP4) expression, alleviating spinal cord edema [97]. In a beta-amyloid (Aβ)-induced toxicity model relevant to Alzheimer’s disease (AD), crocin (150, 300, and 600 nmol) was administered via intra-hippocampal injection. Crocin improved memory and behavioral outcomes. Molecular analyses revealed that crocin slightly modulated autophagy, potentially aiding Aβ clearance, and strongly suppressed apoptosis by restoring the Bax/Bcl-2 balance and reducing caspase-3 activation. It also exhibited anti-inflammatory activity, lowering pro-inflammatory cytokines and NF-κB activation [98].

Evidence from several clinical trials suggests that saffron and its bioactive compounds exhibit neuroprotective effects, making them promising candidates for managing neuropathies. In a randomized, double-anonymized, placebo-controlled clinical trial involving patients with chemotherapy-induced peripheral neuropathy and neuropathic pain, crocin significantly and progressively reduced pain severity, with maximal effects observed during the last three weeks of each treatment phase [99]. In a randomized, double-blind, placebo-controlled trial involving patients with Alzheimer’s disease, saffron supplementation for 12 weeks did not produce additional improvement in global cognitive scores. However, it significantly reduced serum IL-1β and malondialdehyde (MDA) levels, while increasing total antioxidant capacity (TAC) compared to the placebo [100]. Another recent clinical trial reported that supplementation with saffron and its main active compound led to improvements in MDS-UPDRS part II (activities of daily living) and part III (motor function) scores, indicating potential functional benefits [101]. Apart from the clinical observations, in vivo supplementation of safranal (100, 200, or 400 mg/kg) in mice with PTZ-induced epileptic seizures reduced seizure severity, EEG hyperactivity, neuronal hyperexcitability, and hippocampal damage. The antiepileptic effects were mediated by inactivation of GSK-3β via Ser9 phosphorylation, resulting in suppression of the NF-κB inflammatory pathway and mitochondrial-dependent apoptosis [102]. Collectively, the available evidence indicates that saffron holds promise as a potential therapeutic agent for a wide range of neuropathies.

**Table 3:** Comprehensive summary on the protective effects of saffron against neuropathies.

Treatment	Methods of Analysis	Major Findings	References
Crocin (50 mg/kg, i.p.)	PTSD (3 consecutive shocks 0.6 mA, 3 s, along with a sound: 75 dB for 3 s)	Crocin+ extinction learning: ↓ PTSD-like behavior freezing; ↑ BDNF; ↑ pain threshold.	[88]
crocin (10, 30, and 50 mg/kg, i.p.)	Maternal Social isolation from PND 30 to 80 (50 days)	Crocin (30 and 50 mg/kg) ↑ (GSK-3beta) in the hippocampus; ↑ locomotion; enhance memory; ↓ anxiety and depressive-like behaviors; ↓ GSK-3beta level	[89]
Crocin (0.1 to 100 μM)	MPP+-induced apoptosis to PC12 cells	Inhibition of MPP+ mitochondrial dysfunction; ↓ ER stress by regulation of CHOP-Wnt pathway, ↓ apoptosis induced by MPP+	[103]
crocin (30 mg/kg, i.p.)	Repetitive mild traumatic brain injury (rmTBI)	↓ cytokine IL-6; ↑ anti-apoptotic cytokine IL-10; ↓caspase3, Bax and P53; ↑ mRNA levels of Bcl-2, ↑ Nrf2; ↑ HO-1 and NQO-1; ↓ NF-κB	[90]
Crocin (10, 20, and 30 mg/kg by oral gavage) for 4 weeks	Unpredictable chronic mild stress (UCMS) induced anxiety and depression in rats	↓ Serum corticosterone levels, MDA, TNF-α, IL-6; ↑ IL-10, SOD, CAT, Thiols, ↑ BDNF	[91]
C. sativus (50 mg/kg by oral gavage)	Lead (Pb) induced neurotoxicity in Meriones strains	↓ Tyrosine Hydroxylase (TH) to normal, restore locomotor activity by 90%	[92]
Crocin tablet (30 mg/day, 8 weeks)	chemotherapy-induced peripheral neuropathy (CIPN)	↓ Grade of sensory, motor, and neuropathic pain; ↓ adverse effects, lower toxicity	[99]
saffron methanolic extract/crocin-enrichment	Rotenone (ROT)-induced locomotor neurotoxicity in the Drosophila model	↑ GSH.THIOLS; ↓ AChE activity and restore dopamine levels; ↑ life span; ↑ locomotor phenotype	[93]
saffron extract 60 mg/kg by oral gavage	diode laser burns induced ocular hypertension (OHT)	↓ microglion, reversed OHT-induced down-regulation of P2RY12; prevented retinal ganglion cell death in OHT eyes; ↓ neuroinflammation; ↑ intraocular pressure	[104]
Crocin (30 mg/kg/day; i.p., for 30 days)	rotenone (ROT)-induced Parkinson’s Disease in rats	↑ phospho-proline-rich Akt, mTOR and p-p70S6K levels; stimulated PI3K/Akt pathway; ↓ caspase-9, attenuating neurodegeneration (↑ TH and DA); Akt/mTOR activation	[94]
saffron extract (100 or 200 mg/kg, Ip, for 3 weeks)	cerebral ischemia/reperfusion injury (I/R) in rats	↓ MDA, ↓ NO and brain natriuretic peptide (BNP); ↑ GSH; ↓ apoptosis (↓ caspase-3 and Bax protein); ↑ vascular endothelial growth factor	[95]
Safranal (72.5 and 145 mg/kg, ip)	Focal cerebral ischemia/reperfusion injury model	↑ total sulfhydryl (SH); ↓ thiobarbituric acid reactive substances (TBARS); ↓ MDA; ↓ infarct volume and hippocampal cell loss,	[96]
Crocin (20 mg/kg)	Cortical impact induced traumatic brain injury (TBI) in C57BL/6 mice	↑ neurological severity score (NSS); ↓ microglial activation; ↓ cell apoptosis; activation of Notch signaling	[105]
Safranal (25, 50, 100 and 200 mg/kg, ip, for 3 days)	Laminectomy-induced spinal cord injury (SCI) in rats	↑ neurons; anti-apoptotic effect; ↓ inflammation; ↓ expression of AQP-4; ↑ IL-10	[97]
Crocin loaded in SLN (25, 50 mg/kg/day; orally taken for 28 days)	pentylenetetrazol (PTZ) induced oxidative damage	↓ NO; ↑ CAT; ↑ memory; ↓ anxiety; ↓ Nuclear factor kappa B (NF-Κb)	[106]
Crocin (150, 300, and 600 nmol, intra-hippocampal (IH)) (5 mg/mL, i.p.)	amyloid β-induced memory deficit	↑ spatial memory indicators; ↓ Bax/Bcl-2 ratio and cleaved Caspase-3 level; anti-apoptotic effect	[98]
saffron aqueous extract and crocin (15 mg twice daily, 12 weeks)	adult patients with schizophrenia	No serious side effects were observed; ↑ white blood cells	[107]
CSE (10, 15, and 20 mg/kg orally)	scopolamine-induced cognitive impairment, amyloid beta (Aβ) plaque, and neurofibrillary tangles (NFT)	AChE inhibition; ↓ Aβ plaque and NFT	[108]
Stigma of *Crocus sativus*	fly PD model overexpressing several mutant α-synuclein	↑ life span; ameliorate retinal degeneration; ↑ climbing ability in the Drosophila	[109]
Crocin (10, 20, and 30 mg/kg orally) for weeks	Unpredictable chronic mild stress (UCMS) induced Depression and anxiety in rats	↓ corticosterone, ↑ antioxidant defenses, ↓ oxidative damage, ↑ brain-derived neurotrophic factor	[91]
Safron (50 mg/kg) and crocin (30 mg/kg	weight drop model induced (rmTBI)	↓ IFN-γ, TNF-α, MDA, and myeloperoxidase activity (MPO), ↑ GSH ↑ neurological, cognitive, motor, and sensorimotor functions.	[110]
Saffron extract (50 mg/kg, i.p.) for 5 days	Traumatic brain injury (TBI) in the Zebrafish model	anxiolytic effect, prevent fear; ↑ memory performance	[111]
Saffron capsule (15 mg/twice a day) for 12 weeks	Alzheimer’s Disease patients	↓ IL-1β and MDA; ↑ total antioxidant capacity (TAC)	[100]
Safranal (100 mg/kg, 200 mg/kg, or 400 mg/kg)	(PTZ)-induced epileptic seizures in mice	↓ seizure stage; ↓ hyperactivity of neurons; suppressed the NF-κB signaling pathway; ↓ TNF-α and IL-1β	[102]
Crocin (30 mg twice daily)	adult patients with Parkinson’s disease	Enhance daily life activities; attenuate movement disorders	[101]

### 6.6. Anti-Obesity and Anti-Dyslipidemic Effects

Saffron has long held a valued place in traditional medicine, where it has been used for centuries across various cultures as a natural remedy for numerous health issues. Obesity represents a major health risk due to its strong association with serious diseases such as diabetes, cardiovascular disorders, and certain cancers. It is frequently accompanied by lipid imbalances, including elevated levels of cholesterol, LDL, and triglycerides [112]. A study using a zebrafish model concluded that saffron intake reduced body mass index (BMI), supporting its potential as an anti-obesity agent [112]. Numerous studies have also demonstrated the anti-obesity potential of saffron and crocin using high-fat diet–induced obesity models in rats and mice. It was reported by Mashmoul et al. that saffron ethanolic extract or crocin (40 and 80 mg/kg) administered for 8 weeks reduced plasma AST and ALT levels, suggesting protection against liver damage. Histological analysis confirmed hepatoprotection, with normalized liver size, reduced fatty infiltration, and improved tissue structure [113]. Another study focused on the activity of crocin-1 in high-fat diet (HFD)-fed mice, which reduced hepatic triglyceride and cholesterol levels by downregulating lipogenesis-related genes (ACC, Fasn, Srebp1c) and upregulating Peroxisome Proliferator-Activated Receptor Alpha (PPARα), thereby enhancing lipid oxidation [114]. In line with these findings, saffron supplementation was found to be responsible for preventing the development of NAFLD in rats by modulating the gene expression of PPARα [115].

Additionally, saffron at high doses causes faster weight loss and significant reductions in LDL-C, TG, and TC, along with an increase in HDL-C, likely due to crocin and crocetin reducing fat absorption and inhibiting pancreatic lipase [116]. Similar observations have been reported in in vitro studies. At the same time, crocin supplementation to 3T3-L1 preadipocyte cell lines (used as an obesity model) reduced mature adipocyte viability, with minimal effect at 10 μM and the highest inhibition at 50 μM [117]. Another study reported that saffron, specifically crocin and crocetin, significantly reduced lipid droplet accumulation in ADSCs, and that the downregulation of PPARγ, FAS, and GAPDH indicated inhibition of adipocyte differentiation and fatty acid synthesis [118]. Extending this line of evidence, Crocin significantly inhibited adipogenesis-related factors (SREBP-1, CEBPα, PPARγ, FAS) and promoted lipolysis-associated regulators, through AMPK activation [119]. Beyond animal models, clinical evidence has further evaluated the intake of saffron aqueous extract over 8 months. It has been reported that saffron reduced body mass index and suppressed appetite [120]. Overall, evidence from experimental and clinical studies (Table 4) suggests that saffron and its bioactive compounds exert promising anti-obesity effects by modulating appetite, reducing fat accumulation, and enhancing lipid metabolism, making it a potential natural adjunct in weight management.

### 6.7. Anti-Diabetic Effects

Diabetes mellitus is a widespread metabolic disorder ranking among the leading causes of death and disability. Persistent hyperglycemia leads to severe micro- and macrovascular complications, emphasizing the need for adequate glycemic control. Beyond conventional drugs, growing evidence supports the use of herbal remedies [124]. Similar to other herbal products, saffron and its compounds have been reported as promising alternatives to anti-diabetic agents. Several studies documented saffron extract, crocin and safranal improving clinical outcomes (Table 5), in either Streptozotocin, tartrazine or alloxan induced type 2 diabetes in rats, via enhancing insulin secretion and protecting β-cells from degeneration [125], increasing Antioxidant markers (SOD, CAT, GSH) [126], improving obesity, blood glucose [127], lipid profiles [37], alleviate renal fibrosis [128]. These results are further supported by in vitro studies. Drioiche et al. reported that saffron extract exhibited potent inhibitory activity against α-glucosidase and α-amylase, surpassing that of acarbose, suggesting a potential antidiabetic effect [129]. Another study has demonstrated crocetin enhances insulin and GLP-1 while reducing risk [130]. Another in vivo study using a genetic model of type 2 diabetes in mice (db/db) confirmed the hypoglycemic, hypolipidemic, and renal protective effects of crocin (80 mg/kg) for 8 weeks.

Additionally, crocin improved insulin resistance and attenuated pancreatic and renal inflammation by modulating inflammatory cytokines, reducing IL-1β and IL-2, and increasing IL-4 and IL-10, as well as MMP-9 and G-CSF [131]. Clinical trials involving patients with type 1 diabetes mellitus have been conducted to assess the benefits of saffron supplementation. In a Double-Blind, Randomized, Placebo-Controlled Trial on Type 1 diabetes patients, saffron supplementation for 6 months significantly improved serum triglycerides and the diabetes distress score in the active group [132]. These potential mechanisms include antioxidant effects, improved insulin signaling, enhanced GLUT4 translocation, anti-inflammatory actions, and modulation of lipid metabolism. Results align partially with another analysis on saffron’s hypolipidemic and glycemic effects in type 2 diabetes and prediabetes [133]. Outcomes revealed significantly reduced fasting blood sugar (FBS) compared to controls, but no significant changes were observed in triglycerides, HDL, or blood pressure during the intervention [133]. All these reports suggest that saffron and its compounds may serve as a promising therapeutic option against Diabetes.

**Table 5:** Comprehensive summary on the Anti-diabetic potential of saffron.

Treatment	Methods of Analysis	Major Findings	References
Saffron (40 and 80 mg/kg)	Streptozotocin-Induced Diabetes in Rats	↓ blood glucose levels, cholesterol, triglyceride, and LDL; ↑ HDL, SOD, CAT, and GSH; ↓ cognitive deficit	[37]
Saffron (120 mg/kg) for 60 days	Tartrazine-induced diabetic male rats	reduce blood glucose level and creatinine	[127]
Stigma extract (50 mg/kg) for 3 weeks	Streptozotocin-Induced Diabetes in rats	↓ blood glucose levels; ↓ total cholesterol and triglyceride; ↓ urea and creatinine; ↓ AST and ALT	[125]
Crocin (100 mg/kg, i.p.) for 2 weeks	streptozotocin-induced type-2 diabetic rats	↑ serum insulin levels; ↑ (SOD, GSH, and CAT); improve fasting glucose levels	[126]
Crocin solution	Hyperglycemia. In Vivo Evidence from Zebrafish	↓ embryo glucose levels; ↑ insulin expression; ↑ expression of phosphoenolpyruvate carboxykinase 1 (pck1)	[134]
saffron extract	In vitro alpha-amylase and alpha-glucosidase	high inhibitory activity against α-glucosidase and α-amylase	[135]
Saffron extract (84 mg for 6 months)	People with Diabetes Mellitus Type 1	improves serum triglycerides	[132]
Crocin (150 mg/kg orally) 6 weeks	Streptozocin induced type-2 diabetes in rats	↓ plasma TNF-α and IL-1β levels; ↓ pancreas tissue TNF-α and IFN-γ levels; ↓ inflammation and oxidative stress	[136]
Crocin (300 mg/kg in 1 mL PBS) for 8 weeks	Streptozocin induced type-2 diabetes in Sprague Dawley rats	↓ fasting blood glucose levels, ↓ fat accumulation in the liver, alleviate renal fibrosis, and ↓ blood lipid levels	[128]
Saffron extract (0.2–1.2 mg/mL) (400 mg/kg orally)	In vitro inhibition tests of α-amylase and α-glucosidase In vivo Antihyperglycemic Activity in albino Wistar rats	↓ postprandial hyperglycemia; inhibition activity against α-glucosidase and α-amylase	[129]
Crocetin	In vitro biochemical assay In silico	↑ glucose uptake; GPR40/120 agonist; enhance insulin secretion	[130]
Saffron extract capsules twice a day (15 mg) for 8 weeks	A triple-blinded randomized clinical trial	↓ fasting blood sugar	[133]
Crocin (50 mg/kg) for 8 weeks	In vivo investigation of the hypoglycemic effect in db/db mice	↑ insulin and pyruvate kinase	[131]
Saffron extract (15, 200, and 250 mg/kg) for 8 weeks Safranal (15, 20, and 25 mg/kg) for 8 weeks	Alloxan-Induced Diabetes in rats	↓ MDA; ↑ CAT and GSH; ↑ insulin; beta-cells regeneration	[137]

### 6.8. Cardioprotective and Antihypertensive Effects

Among the wide range of natural products investigated for the treatment of prevalent cardiomyopathies, *Crocus sativus* has shown remarkable therapeutic benefits. An increasing number of studies suggest that saffron, particularly crocin and crocetin, has protective effects against a range of cardiomyopathies induced by chemicals, drugs, and heavy metals/pesticides. Isoproterenol is a non-selective β-adrenergic agonist used mainly in research to induce myocardial injury in rats and mice [138,139]. It induces liberation of ROS, leading to an increase in lipid peroxidation [138]. Additionally, it induces disruption of cardiomyocyte membrane integrity, loss of standard myocardial architecture, and triggers a pronounced inflammatory response, characterized by increased pro-inflammatory cytokines (TNF-α, IL-6, IL-1β) and activation of the NF-κB pathway [140]. Persistent injury promotes fibrosis, marked by abnormal collagen deposition and upregulation of fibrogenic mediators, including TGF-β1 and CTGF [140]. Biochemically, myocardial injury is reflected by elevated levels of CK, CK-MB, LDH, and hydroxyproline [141]. As shown in Table 6, treatment with saffron and its bioactive constituents, such as crocetin, crocin, and safranal, has been shown to counteract these pathological changes by attenuating oxidative stress, suppressing inflammation, inhibiting fibrosis, and restoring cardiac biochemical and structural integrity.

In vitro study using LPS to induce cardiomyocyte toxicity in H9c2 cells, supplementation of crocin (10, 20, and 40 µM) increased cell viability, restored thiol content, and dose-dependently suppressed inflammatory cytokines at both protein and gene expression levels. Crocin also reduced COX-2 expression, PGE_2_ production, and iNOS-derived NO generation, limiting peroxynitrite formation and oxidative stress [142]. These findings align with a previous in vitro study, which demonstrated that safranal enhances viability, reduces the release of CK-MB and LDH, attenuates oxidative stress, and inhibits apoptosis in hypoxia/reoxygenation (H/R)-induced myocardial injury using H9c2 cardiomyoblasts [143]. Comparable benefits of safranal (10 µg/mL, 16 h) have been observed in saffron-treated H9c2 cells exposed to doxorubicin and ischemia/reperfusion (I/R) in vitro [144].

In a pilot, randomized, double-anonymized, placebo-controlled clinical trial, a two-month supplementation with crocetin capsules (10 mg/day) demonstrated anti-atherogenic effects, improving HDL levels, LDL/HDL ratio, overall lipid profile, blood pressure, homocysteine concentrations, and physical functioning scores [145]. Apart from this clinical trial, daily oral administration of crocin (10–20 mg/kg, 3 weeks) in the doxorubicin (DOX)-induced cardiotoxicity model reduced myocardial injury biomarkers (AST, troponin T, LDH, CK-MB). Crocin’s benefits involve antioxidant action (Nrf2/HO-1 activation), Ca^2+^ modulation, and anti-apoptotic effects [146]. Similar results were reported by Razmaraii et al. [147], who demonstrated that crocin also protects against endothelial cell injury and apoptosis via caspase inhibition, Bax/Bcl-2 modulation, and suppression of cytochrome c release [147]. Following these results, crocin (40 and 80 mg/kg/day i.p.) significantly attenuated arsenic trioxide-induced cardiotoxicity in Male adult Sprague Dawley rats [148].

Treatment with crocin attenuated particulate matter [149], aluminum phosphide [150], and Diazinon [151]-induced cardiomyopathies by improving hemodynamic parameters, restoring antioxidant enzyme activities, reducing lipid peroxidation, and inflammation. Considering these findings, saffron and its bioactive compounds may serve as an alternative to conventional drugs for protecting against a wide range of heart complications.

**Table 6:** Comprehensive summary on cardioprotective and antihypertensive potential of saffron.

Treatment	Methods of Analysis	Major Findings	References
Crocin (50 or 10 mg/kg/day) for 1 week	Myocardial Infarction Using Sprague−Dawley rats	Lower arrhythmia score; ↑ expression of connexin 43 (Cx43) mRNA	[152]
Crocetin ester (25 and 50 mg/kg) for 14 days	isoproterenol (ISO)-induced acute myocardial ischemia	↓ TNF-α, IL-1β and IL-6; ↓ creatine kinase (CK) and MDA; ↑ SOD; improve histopathological alteration	[138]
Crocin (40 and 80 mg/kg/day i.p.)	Arsenic trioxide-induced cardiotoxicity in rats	↓ ROS; ↑ SOD, GSH and GPx; ↓ MDA, gamma glutamyl transferase and proinflammatory cytokines; ↓ Caspase-3 and Bcl-2	[148]
Crocin (100 and 200 mg/kg/day i.p.) for 14 days	Isoprenaline-induced myocardial fibrosis in mice	↓ IL-6, IL-1, TNF-α and NF-κB, ↓ SOD and CAT; ↓ B cell lymphoma-2, Bcl-2-associated X protein, caspase-3, and cleaved caspase-3 expressions	[140]
Crocin (10 and 20 mg/kg, orally) for 3 weeks.	Doxorubicin-induced myocardial injury in rats	improve ECG profile; restore the balance between pro-and anti-inflammatory cytokines; reduce Cardiac caspase-3 activity	[146]
Crocin (10, 20, and 40 µM) for 1 day	In vitro LPS-induced inflammation in cardiomyocytes	↓ LPS toxicity (↓ TNF-α, PGE2, IL-β, and IL-6); ↑ thiols; ↓ nitric oxide	[142]
Crocin (20 and 40 mg/kg/24 h, for 20 days)	Doxorubicin-induced cardiotoxicity in rats	improved heart damage, structural changes in the myocardium, and ventricular function; it did not affect the in vitro antitumor activity of DOX	[147]
Crocin (3, 30, and 300 µM)	In vitro isolated rat cardiomyocytes	reduce Ca^2+^ flow into cardiomyocytes, resulting in negative inotropic effects on myocardial contractility	[153]
Safranal (0, 10, 20, 40, 80, 160, and 320 μM)	ISO-induced myocardial injury	↓ myocardial apoptosis; downregulating the TNF signaling pathway	[141]
Crocin (12.5, 25, 50 mg/kg/day, i.p.) for 4 weeks	Diazinon induced cardiotoxicity	↓ protein ubiquitylation in heart tissue; ↑ HIF-1α ubiquitylation	[151]
Saffron (5, 10, and 20 mg/kg/day orally) for 21 days	Isoproterenol-induced cardiotoxicity in rats	↑ SOD, GSH, and CAT, ↓ MDA; ↓ left ventricular end-diastolic pressure; ↑ CK-MB and LDH	[139]
crocin (50 mg/kg, i.p.)	PM10-induced cardiotoxicity	Restore Hemodynamic parameters; ↓ MDA and xanthine oxidase XOX; ↑ CAT, SOD, and GPx	[149]
Safranal (10, 30 µM)	(H/R)-induced cardiomyocyte injury in H9c2 cardiac myoblasts	↑ cells viability; ↓ ROS; ↓ CK-MB, LDH, MDA and intracellular Ca2; ↓ caspase-3Bax protein; ↑ Bcl-2 protein; ↑ PI3K/AKT/GSK3β.	[143]
Saffron extract (60 mg/kg/day orally) for 4 weeks	Ischemia-reperfusion induced myocardial injury in Wild Type and ApoE(-/-) mice	↑ eNOS, p-Akt, p-ERK1/2, p-44/p-42 and p-GSK3b-Ser9; ↓ IL-6 and iNOS; ↓ MDA and 3-Nitrotyrosine NT; ↓ infractus size	[154]
Capsule of crocetin (10 mg) for 2 months	pilot, randomized, double-anonymized, placebo-controlled clinical trial	↓ (h-FABP), cellular adhesion molecule 1, vascular cell adhesion molecule 1, monocyte chemoattractant protein 1; ↑ HDL, ↓ systolic and diastolic blood pressure	[145]
Crocetin diamide derivatives (1, 0.2, and 0.04 μM)	In vitro hypoxia-induced injury in H9c2 cells	↑ cell and mitochondrial viability; ↓ LDH	[155]
Crocin (5 and 7 mM)	Aluminum phosphide-induced cardiotoxicity in Human Cardiac Myocyte	↓ Protein carbonyl and MDA; ↑ SOD and CAT	[150]
Safranal (10 μg/mL)	In vitro doxorubicin and ischemia–reperfusion induced cardiomyocyte injury	↓ caspase-3 activity; restored contractile proteins expression; inhibited mitochondrial permeability transition pore	[144]
Safranal (0.075 and 0.025 mL/kg/day orally) for 9 days	Isoprenaline-induced myocardial ischemia in rats	↓ CK, LDH, and MDA; ↑ SOD; ↓ intracellular calcium; improve heart morphology	[156]

### 6.9. Hepatoprotective Effects

The liver plays a vital role in the body due to its central involvement in metabolism and detoxification processes. In addition to these functions, it contributes significantly to immune defense, bile production, and the synthesis of blood-clotting factors [157]. Numerous reports have shown the hepatoprotective potential of saffron and its bioactive compounds. It acts through multiple mechanisms, contributing to liver protection by suppressing oxidative damage and lipid peroxidation, enhancing antioxidant enzyme activity, increasing levels of total thiols and glutathione (GSH), reducing fat accumulation, and minimizing inflammation and other histopathological changes in liver tissue (Table 7).

Several studies highlighted that some drugs and toxins, such as methotrexate [158], Carbon tetrachloride [159], ethanol [160], Leflunomide [161], cisplatin [162], and acetaminophen [163], cause oxidative stress in cells, leading to lipid peroxidation, shown by high MDA levels in rats. These agents also increase serum levels of ALT, AST, and ALP, and are associated with inflammation and histopathological liver lesions. Nevertheless, treatment with saffron extracts or its compounds restored antioxidant defenses, reduced lipid peroxidation, and lowered inflammatory cytokines. Histological analysis also confirmed the hepatoprotective effect, with reduced cellular damage.

Abdu et al. conducted research showing that crocin, in vitro, inhibited proliferation and induced apoptosis in HepG2 liver cancer cells, mainly through cell cycle arrest at the G0 phase [164].

Fatty liver, also known as non-alcoholic fatty liver disease (NAFLD), is a clinical syndrome characterized by lipid accumulation within hepatocytes, steatosis, and infiltration of inflammatory cells into the liver tissue [165]. In vivo, NAFLD was induced in mice by a high-fat diet, and treatment with crocetin improved serum TC, TG, ALT, AST, and liver index. Histopathology confirmed reductions in steatosis, inflammation, and hepatocyte ballooning [165]. Kheirandish et al. reviewed the potential of saffron in preventing or curing hepatic injury. They reported that supplementation with saffron (40 and 50 mg/kg) for 30 days reduced hepatic and renal degenerative changes induced by oxymetholone [166].

**Table 7:** Comprehensive summary on the hepatoprotective effects of saffron.

Treatment	Methods of Analysis	Major Findings	References
Crocin (25 and 50 mg/kg orally)	methotrexate-induced liver injury	↓ MDA, NO, IL-1β, and d TNF-α; ↑ CAT, SOD, GSH, GPx	[158]
Crocin (20, 40, and 80 mg/kg orally) for 8 weeks	Carbone tetrachloride-induced liver fibrosis	↓ nuclear factor-kappa B, IL-6, TNF-α, transforming growth factor β, and α-smooth muscle actin; ↓ caspase 3/7; ↑ peroxisome proliferator-activated receptor γ (PPAR-γ)	[159]
Crocin (30 mg/kg) for 14 days	Copper oxide nanoparticles induced hepatic disturbances	↓ hepatic enzymes activities; ↓ inflammatory biomarkers; repair hepatic alteration	[167]
Crocin (10, 20, and 40 mg/kg i.p.) for 4 weeks	Ethanol toxicity in the rat	↓ MDA; ↑ GSH; restore TNF-α and IL-6 levels; prevent apoptosis	[160]
Saffron extract (60 mg/kg i.p.) for 30 days	Copper Nanoparticles Induced hepatoxicity in mice	↓ MDA, AST, ALT, and ALP; ↑ total antioxidant capacity; partial protection against necrotic cells	[168]
Saffron extract by the gastric tube	Silver Nanoparticles caused Hepatotoxicity	↓ MDA, AST, ALT, and ALP; ↑ GSH	[169]
Crocetin (10, 30, and 50 mg/kg orally)	Non-alcoholic fatty liver disease (NAFLD) in mice	↓ AST, ALT, TC, TG, MDA, CR, UA; ↑ SOD and CAT; suppressed high-fat diet; ↓ TNF-α, IL-6, and IL-1β; ↑ HO-1 and Nrf2	[165]
Crocin (20 mg/kg, orally) for 8 weeks	Leflunomide-induced liver injury	↓ AST, ALT, ALP, hepatic MDA, nitrite, mTOR gene, PI3K gene, TGF-β; ↑ albumin, total protein, hepatic catalase, and GSH	[161]
Safranal (0.025, 0.05 and 0.1 mL/kg/day i.p.) for 14 days	acetaminophen-induced hepatotoxicity in rats	↓ MDA, IL-6, TNF-α, IL-1β; ↑ GSH, GPx and CAT; ↓ AST, ALT and ALP	[163]
Saffron phenolic-enriched fraction nanofibers loaded with C. sativus phenolic	cisplatin-induced hepatotoxicity in mice	↑ weight; ↓ liver enzymes; ↑ GPx, SOD, CAT; ↓ iNOS and IFN-γ	[162]
Saffron, crocin, and safranal (100 mg/kg/day orally) for 7 days	CCL4-Induced Liver Damage	removed histological abnormalities, including necrosis, showing liver injury.	[170]
Saffron (150 and 300 mg/kg/day orally) for 28 days	acetaminophen-induced hepatotoxicity	↓ AST, ALT, ALP, LDH; FXR up-regulation by saffron	[171]
Saffron (40 mg/kg/day orally) for 30 days	Oxymetholone-Induced Hepatic Injury in Rats	↓ in hepatic degenerative changes	[166]
Saffron (80 mg/kg i.p.) 10 days	methotrexate-induced liver toxicity in rats	↓ AST, ALT, ALP, and LDH; ↓ MDA and nitric oxide; ameliorate morphological alterations	[172]
Crocin (50 mg/kg/day orally), (100 µM–300 µM)	In Vivo & In Vitro hepatocellular Carcinoma	↓ C-reactive protein CRP; IL-6; LDH ↓ TNFα, p53, VEGF and NF-κB; anti-tumor effect on HepG2 cells	[164]
Stigma extract (50 mg/kg/day) for 14 days	Carbon tetrachloride induced acute liver injury in rats	↓ AST, ALT, ALP, LDH, creatinine, and MDA; prevent body loss.	[173]
Crocin (50, 100, and 250 mg/kg)	sepsis-induced injury in the liver, kidney, and lungs	↓ IL-1b, TNF-a, IL-6 and IL-10; suppressed p38 MAPK phosphorylation, NF-jB/IjBa and Bcl-2/Bax activation	

### 6.10. Pulmonary Protective Effects

Aromatic plants have long been used as remedies for pulmonary injuries, asthma, and other lung complications. Among them, saffron (*Crocus sativus*) has traditionally been recognized for its therapeutic properties in respiratory conditions. Recent scientific studies now support this traditional use, highlighting saffron’s high potential in improving and alleviating lung-related disorders, as presented in Table 8.

Asthma is a chronic inflammatory disease characterized by airway hyperresponsiveness and infiltration of immune cells [174]. A study combining saffron extract and salbutamol found that their combination improved body weight and hematological profiles, and reduced protein and albumin levels associated with tissue damage. It also downregulated key pro-inflammatory cytokines (IL-4, IL-13, TNF-α, IL-1β) and mitigated epithelial shedding and lung tissue injury, confirming its potent anti-inflammatory and protective effects [174].

Rachad et al. reported that intraperitoneal administration was more effective than oral administration in acrolein-induced lung injury in albino rats. Crocin reduced oxidative and inflammatory markers, restored GSH levels, improved antioxidant defense, and decreased apoptosis [175]. Histopathology confirmed lung tissue protection and reduced fibrosis in Crocin-treated groups [175]. Another observation reported showing that crocin is considered a potential adjunct therapy for oxidative stress-related pulmonary diseases [176]. In line with these results, a study investigated the role of crocin in bleomycin-induced pulmonary fibrosis. Crocin administration (25 mg/kg orally) for 28 days effectively reduced oxidative stress markers, and histologically, it attenuated collagen accumulation and inflammatory infiltration [177].

Lipopolysaccharide (LPS), a major endotoxin found in Gram-negative bacteria, is widely recognized as the primary trigger of acute lung injury [178].In this context, crocin was found to suppress histopathological damage in lung tissue, including alveolar destruction, hemorrhage, and inflammation. The study focused on the inflammatory pathway involving HMGB1 and TLR4, which are known to mediate cytokine release and amplify inflammation. Crocin administration markedly suppressed their expression [178].

Improvement of exercise tolerance, a reduction in serum total oxidant status (TOS), and NF-κB levels were reported in a clinical study [177] following treatment with crocin. In line with the present study, Crocin supplementation over 12 weeks improved pulmonary function tests (PFTs) and significantly increased the 6-min walk distance (6MWD) in patients with COPD [60].

Another study investigated the combined effects of crocin and mesenchymal stem cell-conditioned medium (MSCs-CM) on oxidative stress induced by 2-chloroethyl ethyl sulfide. It revealed that their combination significantly decreases levels of pyruvic acid, aspartic acid, and glutamic acid by enhancing mitochondrial function, reducing oxidative stress, and restoring normal metabolic balance [179]. The study also linked altered serine metabolism with lung fibrosis, which was modulated by crocin and MSCs [179].

In addition to crocin’s protective effects, safranal also demonstrated therapeutic potential.

Safranal also demonstrated therapeutic potential. A study included in vitro human bronchial epithelial cells treated with pro-inflammatory cytokines to induce iNOS and NO production. Safranal significantly reduced NO, peroxynitrite levels, DNA damage, and apoptosis, while restoring mitochondrial function [180]. In vivo, a murine model of the ovalbumin-induced allergic asthma was employed; oral safranal administration resulted in decreased iNOS expression, airway inflammation, epithelial damage, and airway hyperresponsiveness [180]. In line with previous reports, another investigation showed that saffron (20 and 80 mg/kg/day) administered to rats via inhalation attenuated paraquat-induced lung injury, improved antioxidant markers (SOD, CAT, thiol), reduced MDA levels, and alleviated inflammation and tissue injury [181]. Together, all these compelling pieces of evidence suggest that *Crocus sativus* stigmas and their main active compounds are likely to be attractive as a promising therapeutic option against a wide range of pneumopathies.

**Table 8:** Comprehensive summary on the pulmonary potential of saffron.

Treatment	Methods of Analysis	Major Findings	References
Crocin (30 and 60 mg/kg i.p.)	Ovalbumin-sensitized lung tissue in mice	↓ inflammatory cells (eosinophils, neutrophils, macrophages, and lymphocytes); ↓ Drp1, Pgc1α, and Nrf1 levels; ↑ Mfn2	[174]
Crocin (60 mg/kg orally and 50 mg/kg i.p.) for 2 weeks	acrolein-induced lung injury in albino rats	i.p. crocin ↓ MDA, TNF-α, IL-6, Protein carbonyls, 8-hydroxydeoxy guanosine levels; ↑ GSH	[175]
Crocin (7.5, 10, 30 mg/kg)	monocrotaline-induced pulmonary arterial hypertension	↑ Oxidation resistance 1 and P21 gene expression; ↑SOD, GPx, CAT, TAC	[176]
Crocin (25 mg/kg orally) for 28 days	Bleomycin-induced pulmonary fibrosis	↓ TNF-α, MDA, and NO in lungs; ↑ GSH, CAT, GPx	[177]
Crocin (30 and 60 mg/kg)	allergic airway inflammation	Prevent increase in white blood cell; ↓ NF-kB and IL-17; upregulating Nrf2/HO-1 mRNA	[53]
Crocin (50 mg/kg i.p.) for 9 days	LPS-induced acute lung injury	↓ Hemorrhage, inhibition of the HMGB1/TLR4 pathway	[178]
Crocin (30 mg/day) for 12 weeks	A Randomized, Double-Blind, Placebo-Controlled Trial	↓ TOS and NF-κB; ↑ total antioxidant capacity (TAOC); improvement in patients’ exercise capacity	[182]
Crocin (15 mg twice a day) for 12 weeks	A Randomized, Double-Blind, Placebo-controlled trial	↑ pulmonary function tests and walking distance test (6MWD); ↓ TNF-α	[60]
Crocin (25 μM) for 48 h	Oxidative stress induced by 2-chloroethyl ethyl sulfide	decline the injury; reduce inflammation and ROS production; ↑ cell survival	[179]
Safranal (1 and 10 mg/kg) (10 and 100 ng/mL)	In vivo mouse model of Asthma In vitro cytokines induced stress in bronchial epithelial cells	↓ NO, iNOS levels, peroxynitrite ion generation and cytochrome c; ↓ airway hyper-responsiveness and airway cellular infiltration to the lungs; ↓ Th2 type cytokine	[180]
C. sativus extract (20 and 80 mg/kg/day)	Paraquat-induced lung inflammation in rats	↑ IFN-γ, IL-10, SOD, CAT, thiol and EC50; ↓ BALF and MDA; ↓ total and differential WBC	[181]
Saffron extract (150 and 600 mg/kg/day orally) for 15 days	Paraquat-induced lung injuries	↑ SOD, CAT, Thiols in bronchoalveolar lavage fluid (BALF); ↓ TNF-α, IL-10, and tracheal responsiveness	[183]
C. sativus extract (30 mg/kg and 60 mg/kg i.p.) for 28 days	Ovalbumin (OVA)-induced asthma in rats	↓ Bronchoalveolar Lavage Fluid; ↓ total protein and albumin in serum, BALF, and lung tissues; ↓ TNF-α, IL-1β, IL-4, IL-13	[184]

### 6.11. Gastrointestinal Protective Effects

Digestive diseases are common worldwide, significantly affecting health, quality of life, and placing a burden on healthcare systems. Among the many contributing factors are drug-related side effects, microbial infections, and ulcerative conditions that compromise gastrointestinal function. Natural compounds, such as saffron, have been shown to have a protective role by alleviating gastrointestinal disturbances caused by various pathological factors, as illustrated in Table 9.

Recently, an in vitro study by Ouahhoud and colleagues assessed the myorelaxant and antispasmodic effects of saffron on isolated rabbit and rat jejunum. It has been found that saffron extract has moderate myorelaxant and antispasmodic effects on rabbit jejunum contractions; it also slightly reduced spontaneous contractions, suggesting limited smooth muscle relaxant potential [185]. Another report found that an Intraperitoneal injection of crocetin (100 and 200 mg/kg) after a burn injury enhanced key antioxidant enzymes (SOD, CAT, GPx) and lowered MDA levels, indicating reduced lipid peroxidation. The compound also demonstrated anti-inflammatory effects by lowering TNF-α, IL-6, and NF-κB expression. It also decreased intestinal permeability and histological damage [186].

Indomethacin, a non-steroidal anti-inflammatory drug (NSAIDs), is widely used for its analgesic, anti-inflammatory, and antipyretic properties. However, a major side-effect of its use is the frequent occurrence of gastric mucosal injury, which can progress into ulcers and lead to severe complications like bleeding or perforation if left untreated. Conventional treatments such as proton pump inhibitors, H2 receptor antagonists, and antibiotics for H. pylori are widely used to manage these ulcers. While generally effective, these therapies can cause undesirable side effects, limiting their long-term use [187,188]. Natural products such as saffron could be a better option as they have minimal or no adverse effects. Several studies have demonstrated the protective effects of saffron constituents, particularly crocin and safranal, against non-steroidal anti-inflammatory drug (NSAID)-induced gastrointestinal injuries. An Intraperitoneal administration of Crocin (2.50, 10.00, and 40.00 mg/kg) significantly attenuated minor intestinal damage caused by indomethacin in rats by reducing intestinal bleeding, oxidative stress (OSI), inflammatory markers (TNF-α), and apoptotic indicators (caspase-3), while restoring antioxidant enzyme activity (SOD) and histological integrity. Notably, ranitidine alone failed to prevent intestinal lesions, but its combination with high doses of crocin resulted in a synergistic protective effect [187]. Another investigation showed that safranal mitigated gastric ulcers induced by indomethacin through modulation of acid secretion, enhancement of gastric pH, and normalization of oxidative and inflammatory parameters [188]. It also exhibited anti-Helicobacter pylori activity and contributed to mucosal protection, demonstrating efficacy comparable to lansoprazole [188]. Additionally, crocin’s gastroprotective potential was confirmed in a separate study, where it significantly reduced indomethacin-induced gastric injury, oxidative stress (as measured by MDA), and the expression of iNOS and caspase-3. Though crocin did not normalize COX-1 or gastric pH like pantoprazole, it preserved gastric mucus and enhanced COX-2 expression, indicating a distinct but effective protective mechanism [189].

Extracts of saffron and its bioactive compounds are effective in improving clinical outcomes in various models of gastrointestinal injury in rats and mice, including those induced by ethanol, acetic acid, dextran sulfate sodium, and acrylamide through the mechanism lowered MDA levels in gastric tissue [190], mucin-stimulating mechanisms, largely via enhanced PGE2 production [191], reducing pro-inflammatory M1 macrophages and increasing anti-inflammatory M2 macrophages and IL-10+ dendritic cells [192], improving histopathological features [193].

Another study reported that crocin acts as a gastroprotective agent, demonstrating its ability to inhibit apoptosis by reducing caspase-3 expression. Additionally, crocin significantly reduced the total lesion surface area and suppressed the overexpression of iNOS [194]. Collectively, these findings highlight saffron and its compounds as promising natural agents for the prevention and management of gastroenteropathies (Table 9).

**Table 9:** Comprehensive summary on the gastrointestinal protective potential of saffron.

Treatment	Methods of Analysis	Major Findings	References
Stigmas extract (0.3 to 10 mg/mL)	In Vitro Assessment of Myorelaxant and Antispasmodic Effects	Dose-dependent antispasmodic and myorelaxant activity;	[185]
crocin (7.5, 15, or 30 mg/kg, i.p.)	Indomethacin-induced gastric lesions in rats	↓ caspase-3 levels and Inos protein expression; Decrease MDA levels and mucus content	[189]
Crocin (50 mg/kg/day, i.p.) for 3 days	Ethanol-induced gastric injury in rats	↑ gastric juice mucin and mucosal prostaglandin E2 (PGE2), IL-6, TNF-α, myeloperoxidase activity; ↑ SOD and glutathione; ↓ caspase-3 activity and mitigated DNA fragmentation	[191]
Safranal (0.063, 0.25, and 1 mg/kg) for 7 days	Indomethacin-induced gastric ulcer	Ameliorate histological changes and tissue biochemical alterations (↑ SOD and TAC, ↓ MDA, TNF-α, and caspase-3); reduce gastric mucosa lesions.	[188]
crocin (2.50, 10.00 and 40.00 mg/kg i.p.)	Indomethacin induced intestinal ulcer.	Down-regulate intestine weight and organo-somatic index; (↑ SOD; ↓ caspase-3, TNF-α and MDA	[187]
Saffron extract (7.5, 15, 20 and 25 mg/kg orally) for 11 days	DSS induced Colitis in C57BL/6 mice	restore body weight, colon length, histology score; ↓ pro-inflammatory macrophages (M1); ↓ anti-inflammatory macrophages (M2) and IL-10	[192]
Saffron extract (100 and 200 mg/kg orally) for 12 days	Acetic Acid-Induced Gastric Ulcer in male Wistar Rats	Reduce prostaglandin E2 (PGE2) and vascular endothelial growth factor (VEGF), ↓ MDA	[190]
Crocin (20 mg/kg orally) for 8 days	Acetic Acid-Induced Gastric Ulcer in male Sprague Dawley Rats	↓ TNF-α, Ca^2+^ contents, LDH, CRP and Inflammatory cells; Enhance Nrf2 and HO-1 signaling and down-regulate caspase-3 activity; ↑ SOD, GSH and CAT;	[195]
Crocin (15 mg/kg, i.p.) for 30 min before induction of injury	Ischemia-reperfusion-induced gastric injury in rats	Decrease area of gastric lesions; ↓ caspase-3 and iNOS	[194]
Crocetin (100 and 200 mg/kg)	In vitro burn-induced intestinal injury	↑ antioxidants enzymes; reduce inflammatory response (IL-6, TNF-α); improve intestinal permeability, and histological changes	[186]
Crocin (100 mg/kg/day orally) for 15 days	CCL-4 mediated oxidative stress in rats	Reduce histological lesions; ↓ TOS and MDA; ↑ GSH and TAS	[196]
Saffron aqueous extract (10 and 20 mg/kg orally) for 11 days	Dextran sulfate sodium (DSS)-induced colitis in mice	Suppress NF-κB (↓ TNF-α, IL-1β, and IL-6); regulate the composition of gut microbiota; diminishes the susceptibility to colitis reformulate	[197]
Crocin (50 mg/kg orally) for 21 days	Acrylamide induced small and large intestine damage in Wistar rats	↑ GSH and TAS; ↓ MDA, TAS, SOD, and CAT; recover histological damage	[193]
Crocetin (10 and 40 mg/kg orally) for 21 days	DSS-Induced Colitis in mice	Promote inflammation, prolong recovery time from colitis, and disturb gut microbiota composition.	[198]
Safranal (8 and 16 mM)	DSS and Erwinia carotovora 15 (Ecc15) induced intestinal injury in Drosophila	maintain intestinal homeostasis; ↓ antimicrobial peptide (AMP) and ROS; increase the viability of intestinal epithelial cells	[199]
Saffron (50 mg twice daily) for 8 weeks	Open-label, single-center pilot clinical trial	Reduce abdominal pain, diarrhea and rectal bleeding; ↑ IL-10, ↓ TNF-α, IFN-γ, IL-6, IL-2, IL-17A	[200]

### 6.12. Effects on the Reproductive System

Male reproductive health is commonly evaluated through parameters such as serum testosterone concentration, sperm quality (including motility, morphology, and count), and overall semen integrity.

Numerous environmental and physiological conditions can disrupt reproductive hormones and impair semen parameters by increasing oxidative stress. In this context, natural antioxidants like saffron, rich in bioactive compounds such as crocin, crocetin, and safranal, hold promise for protecting and supporting reproductive health. Crocin has been found to improve outcomes by reducing doxorubicin toxicity through a mechanism that involves attenuating oxidative stress by decreasing protein carbonyl levels and increasing key antioxidant markers, such as glutathione peroxidase (GPx) and total antioxidant capacity (TAC) [201]. Cisplatin (CP) is one of the most commonly used anticancer drugs, widely prescribed in clinical settings as a chemotherapeutic agent to treat different types of solid tumors. Like many other chemotherapy drugs, it is associated with severe side effects [202,203,204]. As a natural pharmacological agent, crocin showed a partial protection against CP-induced testicular damage. For example, crocin administration (200 mg/kg i.p.) for 4 days can help by improving testosterone levels, reducing Caspase-3 expression, and restoring testicular histology [204]. Another observation reported a dose-dependent activity of crocin, where lower doses (6.25–25 mg/kg) were less effective against cisplatin. In contrast, the higher dose (100 mg/kg) significantly reduced MDA levels, enhanced antioxidant enzyme activity, and led to histological improvements, including preservation of the germinal layers and spermatogenesis, which were observed only at the highest dose [203].

Azoospermia is a condition in men where no sperm are found in the ejaculate, making natural conception impossible. It affects about 1% of the male population and accounts for up to 15% of male infertility cases. It can be caused by genetic factors, hormonal imbalances, or damage from treatments like chemotherapy [205]. A study reported that the combination of crocin and letrozole improved sperm motility and testosterone levels in rats with busulfan-induced azoospermia, although sperm viability remained unchanged. Crocin also enhanced antioxidant enzyme activity, reduced oxidative stress, and modulated calcium and cAMP signaling pathways to support motility [205].

Consistent with the effects of crocin, the most abundant molecule in saffron stigmas, on male fertility and/or reproduction, an administration of Crocin (200 mg/kg/day, i.p. 14 days) in rats significantly reduced the damage caused by bisphenol A. It boosts antioxidant defenses and reduces oxidative markers. Additionally, Crocin reduced apoptosis by lowering caspase-3 expression in testicular cells. It also helped restore FSH, LH, and testosterone levels [206].

In addition, crocin demonstrated significant protective effects by enhancing sperm quality and counteracting nicotine-induced damage. It improved sperm count, motility, and viability [207]. Crocin also modulated reproductive hormone levels, including testosterone, and had a positive effect on testicular tissue integrity [207].

Testicular torsion is a medical emergency that can lead to ischemia–reperfusion (I/R) injury, causing oxidative stress, cellular damage, and impaired fertility [208]. Crocin, especially at a dose of 100 mg/kg, significantly improved testicular weight, testosterone levels, and antioxidant enzyme activity (SOD, GPx), while reducing MDA levels. Although it did not restore sperm motility or count, it improved sperm viability and testicular histopathology, including the structure of the seminiferous tubules. Crocin also reduced inflammation, protected Leydig cells, and modulated calcium influx and apoptotic pathways [208].

A study investigated the impact of crocin on testicular function in healthy mice. It was found that a high dose of 100 mg/kg, unlike 4 and 20 mg/kg, which were relatively safer, may have adverse effects on the reproductive system [209]. Although high doses may disrupt normal testicular function, crocin has shown a protective impact in toxic conditions due to its antioxidant properties [201,209]. Apart from the clinical observations, in vitro treatment of crocetin (50 µM) to pubertal mouse testis tissue after exposure to 2 Gy X-rays caused significant structural damage and altered the expression of several key stress and DNA repair markers. Pre- and post-treatment with 50 µM crocetin helped reduce tissue injury and oxidative stress [210].

The previously mentioned studies have demonstrated that saffron plays a significant role in enhancing fertility and reproductive health. Overall effects are compiled in Table 10. However, more studies should be conducted to determine the relative safety and efficacy of black cumin for its possible clinical application.

### 6.13. Protection Against Skin Diseases

Saffron-derived compounds have been reported to exhibit strong antioxidant properties, which may protect against oxidative damage. For example, crocetin, a carotenoid derived from saffron, exhibits promising skin-protective effects due to its antioxidant and anti-tyrosinase properties. In vitro studies with B16F10 melanoma cells revealed that crocetin effectively decreased melanin production, mushroom tyrosinase activity, and ROS levels, all while exhibiting no cytotoxic effects [215]. Crocin, a key bioactive compound from saffron stigmas, showed protection of dermal fibroblasts against UVB. Crocin treatment caused a decrease in ROS levels, restored cell proliferation, and enhanced extracellular matrix production. Most importantly, crocin demonstrated anti-aging activity without inducing apoptosis [216].

Saffron extract was shown to confer skin protection in lipopolysaccharide-stimulated RAW 264.7 macrophage cells, effectively reducing the production of reactive oxygen species and nitric oxide. At the same time, it enhanced collagen and hyaluronic acid synthesis in human dermal fibroblasts, supporting skin health and repair [217]. In another study, Safranal, the most crucial volatile compound in saffron, was found to be responsible for inhibiting dermal enzymes that contribute to skin aging. Safranal exhibited notable in vitro antioxidant activity (IC_50_ = 22.7 µg/mL) and effectively inhibited the enzymes elastase, hyaluronidase, and collagenase. The calculated sun protection factor (SPF) was 6.6, also indicating photoprotective potential [218]. Similar outcomes were documented, showing that saffron has senescence-delaying effects in aging mice. It reduced histological signs of aging and alleviated dermal damage, including thinning of collagen fibers and inflammatory infiltration. The treatment preserved skin structure and reduced hemorrhage and ballooning, often observed with age [219]. Since oxidative stress and low-grade inflammation accelerate skin aging, the use of antioxidants becomes essential. Crocin has been found to reduce the expression of key pro-inflammatory mediators (IL-6, IL-8, TNF-α) in keratinocytes, partly by inhibiting the NF-κB pathway, and it effectively scavenges singlet oxygen and hydroxyl radicals, and inhibits UVA-induced peroxidation of squalene and arachidonic acid in skin cells [220].

In skin flap outcomes post-surgery, treatment of saffron extract (40 and 80 mg/kg orally for 7 days) ameliorated flap viability by reducing tissue necrosis, oxidative stress, and apoptotic cell death. As demonstrated in Table 11, these findings suggest that saffron could be clinically valuable in promoting skin flap survival [221].

### 6.14. Bone Regenerative Effects

Numerous studies have demonstrated the in vitro effectiveness of saffron and its active components, crocin and crocetin, in promoting bone repair (Table 12). A treatment with Crocin and crocetin (12.5, 25, 50 µM) demonstrated low cytotoxicity at concentrations below 50 µM. It enhanced the osteogenic differentiation of mesenchymal stem cells after 21 days, via estrogen receptor-related pathways and transcription factors such as Runx2 and ALP [222]. Another study reported similar results showing Crocin boosted bone morphogenetic protein (BMP)-2, vascular endothelial growth factor, and bone formation through improved osteogenic differentiation of Bone MSCs. It also enhanced the production of anti-inflammatory cytokines IL-4 and IL-10, while reducing TNF-α and IL-6 [223]. Moreover, a third study, although conducted in vitro, reported that crocin effectively enhanced the osteogenic differentiation of human bone MSCs by increasing alkaline phosphatase and calcium nodules [224]. In line with these findings, a combination of saffron extract and pulsed electromagnetic fields significantly increases alkaline phosphatase and calcium deposition, thereby enhancing the osteogenic differentiation of BMSCs [225].

Methylglyoxal is a highly reactive compound and a key precursor of advanced glycation end-products (AGEs). It alters bone homeostasis by reducing osteoclast formation, lowering TRAP enzyme activity, promoting oxidative damage, compromising mitochondrial performance, and downregulating glyoxalase I expression. However, like oxidative stress, Crocin counteracts methylglyoxal’s effects by restoring glyoxalase I activity and preventing Methylglyoxal accumulation. It reduces mitochondrial dysfunction, superoxide production, and suppresses collagen degradation [226].

Another study evaluates crocin as a natural pharmacological agent against aluminum chloride-induced cytotoxicity in bone marrow mesenchymal stem cells (BM-MSCs)., e.g., crocin pretreatment enhanced calcium deposition and increased the mRNA expression of Sox-2 and E-cadherin, which are responsible for self-renewal and proliferation [227].

In addition to cell-based assays, several in vivo experiments have been widely used to evaluate bone regenerative potential. Crocin treatment (5 or 10 mg/kg) for 12 weeks preserved bone structure, enhanced ALP and osteocalcin, while reducing bone resorption markers (TRAP and CTX-I). Additionally, crocin lowered pro-inflammatory cytokines (IL-6, TNF-α), decreased oxidative stress in femoral tissues, and improved bone mechanical strength [228]. Another observation in a rat distal femur model demonstrated that crocin reduced the recruitment of immune cells, indicating its potential anti-inflammatory effects [229].

**Table 12:** Comprehensive summary of saffron’s potential in bone regeneration.

Treatment	Methods of Analysis	Major Findings	References
Crocin/crocetin (12.5, 25, 50 µM)	In vitro osteogenic differentiation of mesenchymal stem cells	Improve bone regeneration by increasing the number of mesenchymal stem cells (MSCs) mediated osteoblasts	[222]
Crocin (40 and 80 µM)	Titanium particles induce inflammation and promote osteogenesis	Downregulate Ti particle-induced inflammation by production of anti-inflammatory cytokines (anti-inflammatory (M2) macrophage polarization); improve osteogenic differentiation	[223]
Crocin (10, 20 and 40 mg/kg orally) for 14 days (10, 25 and 50 μmol/L)	In vitro human BMSCs In vivo steroid-induced osteonecrosis of the femoral head	↑ alkaline phosphatase and calcium nodules; Ameliorate osteonecrosis; ↑ expression levels of RUNX2, COL1A1, and OCN in hBMSCs and femoral head tissues; ↓ GSK-3β phosphorylation	[224]
Crocin and bicarbonate de sodium (500 + 80 µg)	Human fetal osteoblast (hFOB) and human osteosarcoma (MG-63) cells; In vivo rat distal femur defects study	Intensify osteoblast proliferation; reduce human osteosarcoma (MG-63) viability by 50%; pro-apoptotic mechanism against osteosarcoma	[229]
Crocin (50, 100, 250 and 500 µM)	AlCl3 induced cytotoxicity in rat Bone Marrow Mesenchymal Stem Cells	↑ mRNA expression of Sox-2 and E-cadherin	[227]
Saffron (800 µg/mL) + Pulsed electromagnetic fields (PEMFs)	In vitro osteogenic differentiation of bone marrow mesenchymal stem cells	↓ dose-dependently, the cell viability, ↑ ALP activity, and synergic effect of saffron and PEMPs on osteogenesis at the initial stage	[225]
Crocin (2.5–5 µM) + nanocurcumin (0.3 and 0.7 µM)	In vitro osteogenic differentiation of bone marrow mesenchymal stem cells	Help MSCs proliferate and protect them from apoptosis; increase the expression of OCT4 and SOX2 genes	[230]
Crocin (2–10 µM) (20–100 mg/mL)	Methylglyoxal-induced osteoclasts in RAW264.7 cell lines	↓ osteoclast function and differentiation and bone resorption; ↓gene expression levels of TRAF6, Akt2, ERK1, OSTM1, and MMP-9; ↓bone resorption activity of osteoclasts	[226]
Crocin (5 or 10 mg/kg orally) for 12 weeks	Metabolic syndrome-induced osteoporosis in rats	↓ tartrate-resistant acid phosphatase and C-terminal telopeptide and TNF-α and IL-6 oxidative stress; protect from histological change in bone; ↑ serum alkaline phosphatase and osteocalcin	[228]

### 6.15. Nephroprotective Effects

Kidney disease is a major health issue in the U.S.; it is the 9th leading cause of death and affects more than 1 in 7 adults, which adds up to around 37 million people [231]. Many chemotherapy drugs can damage the kidneys. This can lead to serious problems like temporary or long-term kidney disease, poor kidney function, or even complete kidney failure [232]. Numerous articles have shown the nephroprotective activity of Saffron and its bioactive molecules.

A study by Hussain and colleagues, using male albino Sprague Dawley rats, has shown amelioration in kidney function through the attenuation of NF-κB mRNA, iNOS, COX2, MDA, and TNF-α, as well as an increase in the antioxidant enzyme SOD, in both the normal and treated groups [232].

Saffron extract administration (100 mg/kg body weight i.p.) is comparable to vitamin E, which is widely used as an antioxidant in terms of protecting the kidney against amikacin-induced nephrotoxicity in rats [233]. A study revealed that methylglyoxal enhances the expression of Nrf2, miR-204, and miR-192, while also increasing the levels of MDA, fasting blood glucose, urine albumin, blood urea nitrogen, and plasma creatinine [234]. However, like oxidative stress, kidney inflammation can be reduced with crocetin. A study involving nephropathic diabetic rats demonstrated that giving 100 mg/kg of crocetin for three months upgrades the metabolic profile. Moreover, it decreased oxidative stress, glycation, and inflammation markers [235]. Gentamicin, a member of the aminoglycoside antibiotic family, is commonly prescribed today to treat infections caused by Gram-negative bacteria [4,236]. Gentamicin tends to accumulate in the proximal tubular cells of the kidneys, leading to a decrease in the glomerular filtration rate [237]. This accumulation also causes a rise in oxidative stress, promotes the production of reactive oxygen species (ROS), and triggers inflammation, cell death, and tissue fibrosis [237,238,239]. Comparable to other natural product stigmas, the extract helped preserve kidney structure and function.

These extracts reduced oxidative stress, inflammation, and lipid peroxidation, as evidenced by lower MDA levels. They also improved histological features by preventing glomerular and tubular damage. Additionally, saffron helped normalize biochemical markers, including creatinine, urea, uric acid, and electrolytes [238]. Another study demonstrates that crocin boosts antioxidant defenses, lowers MDA levels and inflammatory markers, and supports kidney function. These protective effects appear to be linked to the suppression of NF-κB/TLR-4 pathways and the activation of Nrf2 and HO-1, which are involved in cellular defense [237]. Gentamicin also has been found responsible of increase inflammatory markers (NF-κB, TNF-α) and apoptosis-related proteins like caspase-3. Saffron extract significantly reduced these effects by enhancing antioxidant defenses, lowering MDA levels, and downregulating inflammatory pathways, indicating that this natural product can protect against drug-induced kidney damage [239].

Doxorubicin, a well-known anticancer drug, is known to cause severe nephrotoxicity, mainly through oxidative stress and inflammation [232,240]. The increase in MDA NF-κB, TNF-α, iNOS, and COX-2 in renal tissues, leading to structural and functional kidney damage caused by Doxorubicin, is downregulated by the coadministration of crocin, mitigating inflammation [232]. Crocin results show an improvement in kidney structure, and it also downregulates the expression of IL-1β and TGF-β, thereby reducing renal fibrosis [240]. In the rat model (Table 13). Several studies have reported that crocin protects against ischemia/reperfusion injury [241,242]. Saffron has demonstrated protective effects by enhancing renal blood flow, reducing cell injury, and facilitating the restoration of GFR. Additionally, crocin lowers MDA, boosting FRAP, and improving plasma creatinine and urea levels [241]. The potentiality of saffron in preventing kidney damage has also been reviewed elsewhere [243].

## 7. Toxicity of Saffron

Although saffron (*Crocus sativus* L.) is generally recognized as safe when consumed as a food additive, excessive intake or inappropriate use can lead to toxic effects. Its safety profile depends on dosage, duration of exposure, and the physiological condition of the consumer. Several preclinical and clinical studies have been conducted to assess the toxicological effects of saffron and its constituents. Bakshi et al. conducted a study in which the in vitro experiments revealed a higher IC_50_ for normal corneal epithelial cells compared to colon carcinoma cells, indicating lower toxicity toward healthy cells. In the in vivo experiments, administration of a high dose of crocin (150 mg/kg) to tumor-bearing mice showed no reported adverse toxic effects [247]. A recent study on zebrafish examined saffron supplementation at concentrations ranging from 93.75 to 1500 mg/L. Although doses exceeding 5 g/day in humans may lead to adverse effects, the IC_50_ of saffron ethanolic extract in zebrafish was determined to be 1021.374 mg/L, indicating moderate toxicity. At lower tested concentrations, no acute lethal effects were observed [112]. Although saffron is widely recognized for its medicinal benefits, its use during pregnancy raises safety concerns. In the clinical trial on full-term pregnant women, saffron tablets (750 mg within 24 h) improved cervical ripening without causing abnormal uterine contractions or significant adverse maternal or fetal effects. However, the dose and duration were low, and the study excluded high-risk pregnancies. In a double-blind randomized clinical trial, 50 male patients with obsessive compulsive disorder were administered saffron at a dose of 30 mg/day for 10 weeks. The treatment was well tolerated, with no significant adverse effects observed. Biomedical, hormonal, hematological, and urinary parameters showed no significant changes during the study period [248]. Another clinical study reported that a dose of saffron extract (28 mg/day) significantly reduced negative mood and stress-related symptoms, produced a slight improvement in sleep quality, and caused no adverse effects on safety or performance parameters [249].

## 8. Conclusions

Saffron (*Crocus sativus* L.) emerges as a remarkable medicinal plant whose value extends far beyond its culinary and cultural heritage. The accumulated evidence demonstrates that its bioactive constituents, particularly crocin, crocetin, picrocrocin, and safranal, act through multifaceted molecular mechanisms to provide potent antioxidant, anti-inflammatory, immunomodulatory, neuroprotective, cardioprotective, hepatoprotective, antidiabetic, anti-obesity, and anticancer effects. These findings highlight saffron’s potential as a natural therapeutic candidate for the prevention and management of major chronic and degenerative diseases. Moreover, innovations in nanotechnology-based formulations offer new opportunities to overcome limitations related to bioavailability and delivery, thereby reinforcing their relevance in modern pharmacology. Nonetheless, significant challenges remain, including its high production cost, limited global availability, and variability in quality linked to environmental and geographic factors. To fully harness its potential, further translational research and large-scale, well-designed clinical trials are essential. Overall, saffron represents a promising bridge between traditional knowledge and contemporary biomedical science, paving the way for its integration into evidence-based therapeutic strategies.

## Figures and Tables

**Table 1:** Taxonomic classification of *Crocus sativus* L.

Kingdom	Plantae
Division	Magnoliophyta
Class	Liliopsida
Order	Asparagales
Family	Iridaceae
Genus	Crocus
Species	*Crocus sativus*

**Table 2:** Comprehensive summary on the anticancer potential of saffron.

Treatment	Methods of Analysis	Major Findings	References
Crocin and Dimethyl crocetin (22.85 to 0.18 and 11.43 to 0.09 mg/mL)	Glioblastoma A172 and rhabdomyosarcoma TE671cells lines	dose-time dependent cytotoxicity; ↑ regulation of BAX and BID, ↓ regulation of MYCN and BCL-2, SOD1, GSTM1	[66]
Crocin (25, 50, 75, 100, and 125 mg/L)	Cervical carcinoma (HeLa cells)	↓ cell viability	[67]
Safranal (1.6 to 200 µM)	Colon carcinoma colo-205	Cytotoxic effect with an IC50 of 20 µM (↑ ROS and ↓ mitochondrial membrane potential (MMP); ↑ expression of Bax, ↓ Bcl-2; G2/M cell cycle arrest	[68]
cinnamon–saffron extract	oral squamous cell carcinoma SCC-25	Dose-time dependent cytotoxicity; antimigratory effects; ↑ wound healing	[69]
Crocin (1, 20, and 50 μM)	Breast Cancer Cells HCC70, HCC1806, HeLa, and CCD1059sk	Cytotoxic effect in different types of cancer; it inhibited microtubule assembly but induced aggregation of tubulin at higher concentrations; perturbed the mitotic phase in cancerous cells	[70]
Saffron extract (25, 50, 75, and 100 µg/mL)	Cervical cancer cells (HEp-2)	dose-dependent cytotoxicity on (HEp-2); lost the original form and their adherence	[71]
Crocin and picrocrocin (0.5 to 4 mM)	malignant TC-1 and non-malignant COS-7 cell lines	Stimulates apoptosis and prevents cell growth (crocin is more cytotoxic than picrocrocin)	[72]
SWE, SEE, and Crocetin (0 to 1000 µg/mL)	Human lung carcinoma (A549), breast adenocarcinoma (MCF-7), cervical cancer (HeLa)	Dose-time dependent cytotoxicity only in malignant cells; ↑ LDH	[78]
Crocin (200 mg/kg i.p.) for 7 days	N-Nitroso-N-Methylurea-Induced Breast Cancer in Rats	↓ tumor volumes; down regulation of cyclin D1 and p21Cip1; stimulate cell cycle arrest	[79]
saffron extract and safranal (0.5 mM crocin, 0.15 mM)	Kidney Caki-1 and bladder cancer RT4 and RT112 cell lines	↓ cell viability (mixture of crocin and safranal) of tumoral cells only	[74]
Crocin and safranal (0.05–4 and 0.2–3.2 mM)	Oral Squamous Cell Carcinoma (KB Cell Line)	apoptotic effects in the KB cell line	[80]
Saffron extract (0 to 400 µg/mL)	Lymphoblastic T-cell leukemia (Jurkat cell line)	↓ cell growth in a dose-dependent manner; a mixture of safranal and crocin has a lower IC50	[81]
crocetin (β-D-glucosyl) ester (31 to 1000 mg/mL)	human breast adeno-carcinoma cell model (MCF-7)	↓ cell proliferation; inhibit estrogen receptor alpha	[82]
Saffron extract (0 to 4 mg/mL)	Human Prostate Cancer Cells line MDA-PCa-2b	inhibit cell proliferation by apoptotic pathways in dose-dependent effects; a down-regulation of DNA methyltransferases and DNA repair intermediates in a time-dependent manner	[75]
Saffron extract, Crocin And Safranal	Colon cancer cell lines CT26 and HCT116 In vivo, CT26 cells induced colon cancer in mice	↑ body mass, ↓tumor weight; downregulating the expression of inflammatory factors; saffron enhances immunotherapy efficacy (↑ Th 17 cells differentiation, modulates CD4+/CD8+T ratio); suppresses the proliferation of CT26 and HCT116	[76]
Saffron extract, Crocin And Safranal (up to 8 mg/mL)	human colorectal cancer cell lines	↓ in cellular proliferation (upregulation of caspase 3 and 7); ↓ wound area	[83]
Nanoliposome with saffron in vitro (25 mg/mL) In vivo (75 and 300 mg/kg tail vein injections)	In vitro and in vivo C26 colon carcinoma	Higher cytotoxic effect in the formulation than aqueous extract; nanoliposome reduces tumor volume at 300 mg/kg due to the site-directed drug delivery	[77]
crocetin/dendrimers complex (PPI and PAMAM) (0.8 to 50 µM)	human breast cancer MCF-7 cells	↑ percentage of apoptotic cells in encapsulated crocetin due to higher cellular uptake	[84]
Crocetin/poly (lactic-co-glycolic acid) (PLGA)	human breast cancer MCF-7 cells	Encapsulation increases cytotoxicity; IC50 of PLGA–crocetin NPs was 84.73 µM, free crocetin showed higher IC50 values 589.65 µM	[85]
Saffron and silver/zinc oxide nanocomposites (0 to 8 µg/mL)	HeLa cervical cancer cell line	↑ ROS, ↓mitochondrial membrane potential; ↑ apoptotic signals by reducing Cyclin D, PCNA, and CDK expression;	[86]
Crocin-loaded chitosan-alginate	human breast cancer MCF-7 cells	Crocin reduces viability in a dose- and time-dependent manner.	[87]

**Table 4:** Comprehensive summary on the Anti-Obesity and Anti-Dyslipidemia potential of saffron.

Treatment	Methods of Analysis	Major Findings	References
Stigma extract (93.75–1500 mg/L)	high-fat diet-induced zebrafish model	100 and 200 mg/L ↓ body mass index 1500 mg/L is highly toxic	[112]
Crocin (10–50 μM)	In vitro model of obesity	↓ cell viability of adipocyte; ↓ adipocyte differentiation; ↑ expression levels of AMP-activated protein Kinase	[117]
Saffron extract (1.25, 5, 10, 20, 40, and 80 μg/mL)	In vitro cytotoxicity in adipocyte differentiation in human adipose-derived stem cells (ADSCs)	↓ adipocyte differentiation with no cytotoxicity; ↓ PPARγ, GAPDH, and FAS proteins	[118]
saffron aqueous extract (30 mg), crocin (30 mg) for 8 weeks	Double-blind and placebo-controlled trial on a patient with coronary artery disease	↓ body mass index (BMI), suppresses appetite	[120]
Saffron extract (40 and 80 mg/kg/day)	High-fat diet induced Obesity in rats	↓ plasma glucose levels; ↓ insulin; ↑ adiponectin and ghrelin; ↓ leptine	[113]
Crocin-I (20 mg/kg/day) for 10 weeks orally	high-fat diet (HFD) -induced obese mice	↓ body and liver weight; ↑ glucose resistance; ↓ lipid accumulation in liver; ↓ intestinal microbial disorders; ↓ F/B ratio; repaired altered intestinal barrier functioning; ↓ intestinal inflammation	[114]
Saffron (250 mg/kg) for 7 weeks	high-fat, high-sugar diet in a rat model	↓ body weight and food intake; ↓ insulin; lower total antioxidants activities	[115]
Saffron (20 mg/kg) 20 μM crocin in vitro	Differentiation of adipocytes in vitro/in vivo adipose tissue in db/db mice	inhibits adipogenesis and promotes lipolysis via activation of AMPK	[119]
Saffron extract and crocin (40 and 80 mg/kg) for 8 weeks	High-fat diet-induced obesity rat model	↑ glucose, pyruvate, betaine, and taurine; ↓ lactate, alanine, and creatinine; ↓ Trimethylamine N-oxide	[121]
Crocin (20 mg/kg orally) for 12 weeks	Streptozotocin and high-fat diet-induced obesity and type 2 diabetes in mice	Activation of AMPK (inhibition of adipose formation) inhibits the changes of glucose metabolic parameters and serum lipid profiles in wild-type diabetic mice.	[122]
Saffron extract (40 and 80 mg/kg orally) for 3 weeks	high-fat diet-induced in rats	↓ total cholesterol, triglyceride and LDL; ↑ HDL; ↓ atherosclerosis-index and liver enzymes; ↓ insulin, leptin, resistin; ↑ adiponectin; ↓ MDA	[123]
Saffron extract (40 and 80 mg/kg) for 4 weeks	Insulin-sensitizing adipokine in high-calorie diet rats	↓ body weight, ↑ insulin, and adiponectin	[116]

**Table 10:** Comprehensive summary of saffron’s protective potential on the reproductive system.

Treatment	Methods of Analysis	Major Findings	References
Crocin (10 and 50 mg/kg/day orally) for 8 weeks	Doxorubicin-Induced Testicular Toxicity in Rats	↑ GPx, TAC, and protein carbonyl (PC) levels in testicular tissue	[201]
Crocetin (50 µM)	In vitro Irradiation Injury of the Pubertal Testis	↓ testis injury; did not restore levels of p62 and LC3-II; ↑ SOD2, CAT and HuR; restore PARP1 and PCNA	[210]
Saffron (100 mg/kg orally)	Khat induced testicular dysfunction in male rats	improve in testicular histology, histochemistry, and biochemical results (SOD, CAT, MDA)	[211]
Crocin (12.5, 25 and 50 mg/kg i.p.) 4 weeks	Nicotine-induced damage to reproductive parameters in mice	↑ testosterone, sperm, count, viability, and motility; testis weight	[207]
Crocin (4, 20 and 100 mg/kg orally) for 6 weeks	Crocin impact on spermatogenesis	At high doses ↓, the diameter of seminiferous tubules and the number of sperm in seminiferous tubules are affected by ↓ Sertoli cells.	[209]
Crocin (15, 30 and 60 mg/kg/day orally) for 31 days	Methylglyoxal-induced reproductive system dysfunction in mice	Improve testicular morphology, testosterone, and sperm count; ↓ Luteinizing hormone	[212]
Crocin (15 mg i.p.)	Busulfan-induced Azoospermia in rats	improve sperm motility and testosterone levels, ↑ total sperm count; ↓ Total Oxidant Status (TOS), reduce DNA damage	[205]
Crocin (200 mg/kg i.p.) for 4 days	Cisplatin-induced changes in the testis in rats	Slightly improve body weights, testicular weights, and serum testosterone levels; ↓ apoptotic cells.	[204]
Crocin (50 and 100 mg/kg i.p.)	Ischemia-Reperfusion followed by torsion induced testicular injury	↑ SOD, GPx activity, and testosterone level; ↓ MDA; improve the histopathological parameters	[208]
Crocin (200 mg/kg/day, i.p.) for 14 days	Bisphenol induced testicular toxicity	↓ apoptosis, caspase-3; ↓ TBARS; ↑ sperm motility and sperm count ↑ FSH, LH, and testosterone, ↑ in the P-gp expression	[206]
Crocin (6.25–100 mg/kg i.p.) 16 days	cisplatin-induced testicular impairment in rats	↑ testis weight, testosterone level, SOD; ↓ lipid peroxidation, ↑ germinal layer area	[203]
Crocin (50 mg/kg)	Streptozotocin-Induced Diabetic Rats	Recover testicular tissue damage, restore sperm parameters	[213]
Saffron extract (10 and 20 mg/kg orally) for 30 days	D-gal-induced late-onset hypogonadism	improve Leydig cell resistance and escape apoptosis; ↑ testosterone; modulate the PI3K-Akt-Nrf2 signaling pathway	[214]

**Table 11:** Comprehensive summary of saffron’s protective potential against skin diseases.

Treatment	Methods of Analysis	Major Findings	References
Saffron extract (40 and 80 mg/kg orally) for 7 days	Skin flap surgery in rats	↓ flap necrosis; improve histological healing; ↑ Bax and Bcl-2; ↓ MPO and MDA	[221]
Crocetin (0.5 to 32 µM)	In vitro ROS-scavenging and Anti-tyrosinase Properties of Crocetin	Inhibition of tyrosinase activity; ↓melanin in B16 melanoma cells; ↓ tyrosinase and MITF; ↓ ROS	[215]
Crocin (12.5, 50, 100 µM)	Ultraviolet β-induced dermal fibroblast photoaging	↓ cell proliferation; restore cell cycle arrest; anti-aging (↓ SA-β-gal-positive cells); ↑in Col-1 expression	[216]
Saffron extract (80 mg/kg/day intragastrically)	The anti-aging activity of saffron in old mice	Anti-aging activity by ameliorating the thickness of the skin	[219]
Safranal (2 to 100 µg/mL)	In vitro biochemical assays (dermal enzymes inhibition activities)	Inhibit dermal enzyme (elastase, collagenase, and hyaluronidase)	[218]
Saffron extract (100 and 200 µg/mg)	In vitro biochemical assays (tyrosinase and collagenase inhibition activities)	Inhibit tyrosinase and collagenase; ↓ ROS and NO in macrophage cells; ↑ collagen and hyaluronic acid synthesis; improve wound healing	[217]
Crocin (0.3 mM and 1 mM) (10 to 100 µM)	In vitro peroxidation and inflammation on NHEKs and HDF	↓ release of pro-inflammatory mediators; ↑ antioxidant defense; downregulation of NF-κB signaling pathway in NHEKs	[220]

**Table 13:** Comprehensive summary of saffron’s nephroprotective effects.

Treatment	Methods of Analysis	Major Findings	References
Crocin (100 mg/kg body i.p.) for 3 weeks	Doxorubicin-Induced Nephrotoxicity in rats	↑ kidney functions; upregulate creatinine clearance; ↓ MDA, NF-κB, iNOS, COX2, and TNFα; ↑ SOD; Area of renal proximal and distal convoluted tubules and distal convoluted tubules	[232]
Saffron extract (50 mg/mL orally) for 3 weeks	Amikacin-Induced Nephrotoxicity in Albino Rats	↑ nuclei number; ↓ MDA in renal tissue; ↑ number of intact proximal tubules	[233]
Crocetin (100 mg/kg i.p.) for 3 months	STZ-induced diabetic nephropathy in rats	↓ Proteinuria, Creatinine, glomerulosclerosis, diverse glycation, oxidative stress, and inflammatory markers	[235]
Crocin (15, 30 and 60 mg/kg/day orally) for 2 weeks	methylglyoxal-induced diabetic nephropathy in mice	↓ MDA, proteinuria, blood urea nitrogen, plasma creatinine, and Nrf2, miR-204, miR-192 expression; ↑ SOD, CAT, and glutathione glyoxalase 1	[234]
Crocin (0, 100, 200 and 400 mg/kg i.p.)	Ischemia-reperfusion-induced renal injuries	↓ creatinine, urea-nitrogen, MDA; ↓ leukocyte infiltration; ↓ ICAM-1 and TNF-α mRNA expression levels in a dose-dependent manner	[241]
Crocin (100 mg/kg i.p.) for 30 days	passive Heymann nephritis (PHN) induced by anti-Fx1A antiserum in rats	↓ creatinine, proteinuria, total cholesterol; ↑ in urinary creatinine clearance, ↑ SOD, CAT, GSH; ↓ MDA; activated the Sirt1/Nrf2/HO-1 pathways; attenuated the renal histopathological changes	[244]
Crocin (40 mg/kg i.p.) 14 days	doxorubicin-induced disturbances in the kidney	↑ SOD and CAT, GSH, prevent inflammation and oxidative stress; stabilize cellular redox homeostasis, reducing renal fibrosis	[240]
Stigmas extract (50 mg/kg orally) for 14 days	Gentamicin-Induced Renal Toxicity	Protect against weight loss; ↓ plasma creatinine, ↑ urine creatinine clearance, ↑ plasma potassium levels, ↓ and MDA.	[238]
Crocin (25 and 50 mg/kg i.p.) for 8 days	gentamicin-induced nephrotoxicity in rats	↓ kidney weight; ↑ in uric acid, creatinine, and urea levels; ↓ pro-inflammatory biomarkers; decrease in COX-2, NF-κB, and TLR-4; up-regulate Bcl-2, Nrf-2, and HO-1	[237]
Saffron extract (80 mg/kg i.p.) for 7 days	Gentamicin-Induced Renal Toxicity in Albino Rats	↑ GPx and SOD; ↓ NF-κB, TNF-α, Creatinine, and urea	[239]
Crocin-loaded naniosomes intravenous injection	Ischemia–reperfusion injuries in the rat kidney	Reduce histopathological changes in sick rats; ↑ SOD; ↓ MDA; creatinine and urea.	[242]
Crocin (40 mg/kg, i.p.) for 10 days	Colistin-induced nephrotoxicity in a rat	↓ in BUN and creatinine, ↑ GSH levels, and ameliorated the histopathological alterations	[243]
Saffron extract	Oxymetholone-Induced Hepatic and Renal Injury in Rats	↓ renal degenerative changes	[166]
Crocin (50 mg/kg orally) for 8 weeks	db/db mice	Protect renal structure; ↓ phospho-IkBa and NF-kB; ↑ nuclear respiratory factor 2, manganese SOD 1, heme oxygenase-1, and catalase	[131]
Crocin (0.5 mg/kg orally) for 8 weeks	Vincristine Sulfate Drug-Induced renal toxicity	↓ blood urea nitrogen, creatinine, and uric acid; improvement in serum TAC content.	[245]
Crocin (12.5, 25, and 50 mg/kg i.p.) for 28 days	Methotrexate induced renal toxicity	↓ MDA, creatinine; NO levels; enhance the antioxidant capacity	[246]

## Data Availability

No new data were created or analyzed in this study.

## Abbreviations

Akt: Protein Kinase B. mTOR: Mechanistic Target of Rapamycin. 6MWD: Six-Minute Walk Distance. Aβ: Amyloid-β. ACC: Acetyl-CoA Carboxylase. AChE: Acetylcholinesterase. ADAMTS: A Disintegrin and Metalloproteinase with Thrombospondin Motifs. Akt: Protein Kinase B. ALP: Alkaline Phosphatase. ALT: Alanine Aminotransferase. AMPK: AMP-Activated Protein Kinase. AQP4: Aquaporin-4. AST: Aspartate Aminotransferase. ATP: Adenosine Triphosphate. BALF: Bronchoalveolar Lavage Fluid. Bcl-2: B-cell lymphoma 2. Bax: Bcl-2 Associated X Protein. BDNF: Brain-Derived Neurotrophic Factor. BMI: Body Mass Index. BNP: Brain Natriuretic Peptide. cAMP: Cyclic Adenosine Monophosphate. CAT: Catalase. CBF/CK: Creatine Kinase. CK-MB: Creatine Kinase MB Isoenzyme. COX-2: Cyclooxygenase-2. CP: Cisplatin. CTGF: Connective Tissue Growth Factor. CUPRAC: Cupric Ion Reducing Antioxidant Capacity. DA: Dopamine. DZN: Diazinon. DNA: Deoxyribonucleic Acid. DOX: Doxorubicin. ECG: Electrocardiogram. eNOS: Endothelial Nitric Oxide Synthase. ERK: Extracellular Signal-Regulated Kinase. FAS: Fatty Acid Synthase. FBS: Fasting Blood Sugar. FoxO3: Forkhead Box O3. FRAP: Ferric Reducing Antioxidant Power. FSH: Follicle-Stimulating Hormone. GC-MS: Gas Chromatography–Mass Spectrometry. GLUT4: Glucose Transporter Type 4. GPx: Glutathione Peroxidase. GPR40/120: G-Protein-Coupled Receptor 40/120. GSH: Reduced Glutathione. GST: Glutathione S-Transferase. GSK-3β: Glycogen Synthase Kinase-3 Beta. HFD: High-Fat Diet. HPLC: High-Performance Liquid Chromatography. H/R: Hypoxia/Reoxygenation. h-FABP: Heart-Type Fatty Acid-Binding Protein. HDL: High-Density Lipoprotein. HO-1: Heme Oxygenase-1. IC50: Half-Maximal Inhibitory Concentration. IFN-γ: Interferon-Gamma. IL-1β/IL-4/IL-6/IL-10/IL-17: Interleukin-1β/-4/-6/-10/-17. iNOS: Inducible Nitric Oxide Synthase. I/R: Ischemia/Reperfusion. JAK: Janus Kinase. JNK: c-Jun N-terminal Kinase. LDH: Lactate Dehydrogenase. LDL: Low-Density Lipoprotein. LC-MS: Liquid Chromatography–Mass Spectrometry. LH: Luteinizing Hormone. LPS: Lipopolysaccharide. MAPK: Mitogen-Activated Protein Kinase. MCP-1: Monocyte Chemoattractant Protein-1. MDA—Malondialdehyde. MDS-UPDRS: Movement Disorder Society–Unified Parkinson’s Disease Rating Scale. MMP-1/-3/-13: Matrix Metalloproteinase-1/-3/-13. mTOR: Mechanistic Target of Rapamycin. NAFLD: Non-Alcoholic Fatty Liver Disease. NADH: Nicotinamide Adenine Dinucleotide (reduced). NADPH: Nicotinamide Adenine Dinucleotide Phosphate (reduced). NF-κB: Nuclear Factor kappa-light-chain-enhancer of activated B cells. NLRP3: NOD-Like Receptor Family Pyrin Domain Containing 3. NO: Nitric Oxide. NQO1: NAD(P)H Quinone Dehydrogenase 1. Nrf2: Nuclear Factor Erythroid 2–Related Factor 2. NSAID(s): Non-Steroidal Anti-Inflammatory Drug(s). NSS: Neurological Severity Score. OHT: Ocular Hypertension.). OVA: Ovalbumin. PD-L1: Programmed Death-Ligand 1. PFTs: Pulmonary Function Tests. PGE_2_: Prostaglandin E_2_. PI3K: Phosphoinositide 3-Kinase. PK: Pyruvate Kinase. PLGA: Poly (lactic-co-glycolic acid). PKC: Protein Kinase C. PM10: Particulate Matter < 10 µm. PPARα/PPARγ: Peroxisome Proliferator-Activated Receptor-Alpha/-Gamma. PRAS40: Proline-Rich AKT Substrate of 40 kDa. PTSD: Post-Traumatic Stress Disorder. PTZ: Pentylenetetrazol. RNA: Ribonucleic Acid. ROS: Reactive Oxygen Species. SIRT3: Sirtuin-3. SOD: Superoxide Dismutase. SREBP-1/-1c: Sterol Regulatory Element-Binding Protein-1/-1c. STAT3: Signal Transducer and Activator of Transcription 3. TAC: Total Antioxidant Capacity. TBI: Traumatic Brain Injury. TGF-β: Transforming Growth Factor-Beta. Th17: T helper 17 (cells). TLR4: Toll-Like Receptor 4. TNF-α: Tumor Necrosis Factor-Alpha. UC: Ulcerative Colitis. VCAM-1: Vascular Cell Adhesion Molecule-1. VEGF: Vascular Endothelial Growth Factor.
